# Lymphatic endothelial progenitors originate from plastic myeloid cells activated by toll-like receptor-4

**DOI:** 10.1371/journal.pone.0179257

**Published:** 2017-06-09

**Authors:** Lisa D. Volk-Draper, Kelly L. Hall, Andrew C. Wilber, Sophia Ran

**Affiliations:** 1 Department of Medical Microbiology, Immunology, and Cell Biology, Southern Illinois University School of Medicine, Springfield, IL, United States of America; 2 Simmons Cancer Institute, Southern Illinois University School of Medicine, Springfield, IL, United States of America; Universita degli Studi di Milano, ITALY

## Abstract

**Background:**

Myeloid-derived lymphatic endothelial cells (M-LECP) are induced by inflammation and play an important role in adult lymphangiogenesis. However, the mechanisms driving M-LECP differentiation are currently unclear. We previously showed that activation of Toll-like receptor-4 (TLR4) induces myeloid-lymphatic transition (MLT) of immortalized mouse myeloid cells. Here the goals were to assess the potential of different TLR4 ligands to induce pro-lymphatic reprogramming in human and mouse primary myeloid cells and to identify transcriptional changes regulating this process.

**Methodology/Principal findings:**

Human and mouse myeloid cells were reprogrammed to the lymphatic phenotype by TLR4 ligands including lipopolysaccharide (LPS), recombinant high mobility group box 1 protein (HMGB1), and paclitaxel. TLR4 induced similar MLT in cells from mice of different strains and immune status. Commonly induced genes were detected by transcriptional profiling in human and mouse myeloid cells from either immunocompetent or immunodeficient mice. Shared trends included: (1) novel expression of lymphatic-specific markers vascular endothelial growth factor receptor-3 (VEGFR-3), lymphatic vessel endothelial hyaluronan receptor-1 (LYVE-1) and podoplanin (PDPN) largely absent prior to induction; (2) lack of notable changes in blood vessel-specific markers; (3) transient expression of VEGFR-3, but sustained increase of vascular endothelial growth factor-C (VEGF-C) and a variety of inflammatory cytokines; (4) dependency of VEGFR-3 upregulation and other LEC genes on NF-κB; and (5) novel expression of lymphatic-specific (e.g., PROX1) and stem/progenitor (e.g., E2F1) transcription factors known for their roles in adult and embryonic vascular formation. M-LECP generated by TLR4 ligands *in vitro* were functional *in vivo* as demonstrated by significantly increased lymphatic vessel density and lymphatic metastasis detected in orthotopic breast cancer models.

**Conclusions/Significance:**

We established a novel TLR4-dependent protocol for *in vitro* production of functionally competent M-LECP from primary human or mouse myeloid cells and identified many potential regulators of this process. This information can be further exploited for research and therapeutic purposes.

## Introduction

The lymphatic system plays a key role in physiology to ensure tissue homeostasis, lipid metabolism, and immune defense [1,2]. Abnormalities, due to genetic mutation, disease or surgery, result in lymphedema and associated pathologies due to inadequate tissue perfusion, chronic inflammation, and defective immune response [2]. Tumor-induced lymphangiogenesis is central to metastasis because tumor spread typically begins with lymphatic-assisted cell transport to regional lymph nodes [3,4]. Detailed understanding of the mechanisms underlying lymphangiogenesis, i.e. generation of new lymphatic vessels, is needed to address therapeutic needs of deficient or excessive lymphatic formation. Previous studies established that postnatal lymphangiogenesis is induced by chronic inflammation, tissue injury or cancer [3,5]. Whether this process requires lymphatic endothelial cell progenitors (LECP) remains a subject of debate [6,7]. Clarification of this question would advance our current understanding of lymphatic biology and promote the rational design of therapies intending to control lymphatic formation under pathological conditions.

Two concepts exist to explain mechanisms driving adult lymphangiogenesis. In the first and most widely held view, lymphangiogenesis occurs via sprouting from existing lymphatic vessels following activation of vascular endothelial growth factor receptor 3 (VEGFR-3) on lymphatic endothelial cells (LEC). VEGFR-3 activated by its ligands VEGF-C [8] or VEGF-D [9] promotes LEC division followed by their migration into a matrix-guided shaft and formation of a new sprout from the original “mother” vessel. This concept assumes that postnatal lymphangiogenesis does not require LECP originating from bone marrow (BM)-derived myeloid cells (BMDM) or other avascular sources [6]. It is accepted that BMDM promote lymphatic formation; however, their pro-lymphatic role is thought to be restricted to production of paracrine lymphangiogenic factors such as VEGF-A [10] or VEGF-C [11].

An alternative concept infers that LECP present in tumors [12,13] and other inflamed sites [13–16] play a significant role in lymphatic formation [17,18]. This concept is supported by observations not effectively explained by the canonical view. First, BMDM, putative precursors for lymphatic progenitors, are ubiquitously associated with lymphangiogenesis [19], and density of BMDM at inflamed sites including tumors directly correlates with number of lymphatic vessels [11]. Second, inflammation and tumor-mobilized BMDM often express lymphatic-specific markers such as VEGFR-3 [14,15], LYVE-1 [12,14], and podoplanin (PDPN) [12,13,16]. Expression of LEC markers in myeloid cells that prior to inflammation lack these proteins strongly supports the idea that these cells are lymphatic progenitors derived from myeloid precursors [17]. This notion is also supported by expression of stem/progenitor markers such as CD133 in this cell population [20,21] suggesting their immature status. Third, cells with mixed myeloid-lymphatic identity possess the unique ability to integrate into preexisting lymphatic vessels [16,22], an event that precedes sprouting [13,15,22]. The requirement for structural contribution of LECP to lymphatic vessels cannot be explained by a paracrine induction of lymphangiogenesis, which, by definition, exclusively relies on soluble factors. Fourth, LECP are absent in healthy persons but present at high levels in the blood of cancer patients. Moreover, levels of circulating LECP strongly correlate with disease stage, lymph node metastasis, and patient survival [21,23]. Thus, LECP exist in humans and significantly impact cancer pathology. Finally, LECP can be generated from human or mouse myeloid cells by inflammatory mediators under controlled conditions *in vitro* [12,22,24]. *In vitro* generated LECP possess many LEC properties and have the ability to expand the lymphatic network at inflammatory or tumor sites *in vivo* [12,13,24,25]. Collectively, these studies provide evidence for existence of adult LECP and their role in expanding existing lymphatics under inflammatory conditions including tumors. We expand upon this idea by proposing that either pathogen-related or cancer-induced inflammation causes pro-lymphatic reprogramming of myeloid or hematopoietic precursors followed by recruitment of these cells to inflamed sites or tumors where they promote formation of lymphatic vessels. Because this subset is mainly derived from myeloid cells [15,16,22,26], we refer to it as Myeloid/Monocyte-derived Lymphatic Endothelial Cell Progenitors (M-LECP).

*In vitro* differentiation of myeloid precursors into lymphatic-like cells represents the key evidence supporting the existence and functional significance of M-LECP. Such pro-lymphatic reprogramming has been shown for human monocytes isolated from peripheral or cord blood [24,27], human pluripotent stem cell lines [25], mouse embryonic cells [28], mouse BM-derived CD11b^+^ and mononuclear cells [13,16,29], mouse and human mesenchymal stem cells [30] and adipose-derived stem cells [31]. The main criteria for defining differentiated cells as LECP are as follows: 1) expression of specific LEC markers [16,24,25,27]; 2) acquisition of an endothelial-specific cobblestone morphology and/or ability to form tubes when grown in matrigel [16]; 3) demonstrated function *in vivo* evidenced by integration into lymphatic vessels [12,15,22] and a statistically significant increase in lymphatic vessel density (LVD) in inflammatory and tissue remodeling models [24,25,32]; and 4) evidence for enhanced functionality of new lymphatics such as improved relief from lymphedema [32] and an accelerated rate of healing wounds [25].

While these collective reports solidly support the existence of lymphatic progenitors, many details regarding M-LECP phenotype, inductive stimuli and differentiation mechanisms are currently unclear. The main factors known to induce pro-lymphatic differentiation are VEGF-A and VEGF-C [24,25,27,33]. We previously reported [22] that pro-lymphatic differentiation can also be induced by lipopolysaccharide (LPS), a ligand of Toll-Like Receptor-4 (TLR4). TLR4-mediated generation of lymphatic progenitors is consistent with reported LPS-driven lymphangiogenesis in inflammatory models [22,34] and detection of lymphatic insufficiency in TLR4-null mice [35].

We previously showed TLR4-dependent myeloid-lymphatic transition (MLT) in a mouse immortalized macrophage line, RAW264.7 [22]. Here, we used three structurally unrelated TLR4 ligands to demonstrate MLT of primary human and mouse myeloid cells. Adoptive transfer of M-LECP into mice with peritonitis and orthotopic breast cancer models resulted in their integration into lymphatic vessels followed by increased LVD. We show that TLR4 induces lymphatic reprogramming independently of mouse strain or immune status, and that human and mouse M-LECP generated by TLR4 activation exhibit similar profile of LEC-related genes. Collectively, results of these studies provide insights into development of the LEC lineage in adults, and establish an efficient differentiation protocol for production of human or mouse lymphatic-promoting cells. This new information expands our current understanding of the lymphatic biology and could be instrumental for development of therapeutic approaches to correct the disorders of the lymphatic system.

## Materials and methods

### Human blood collection

Human venous blood samples were purchased from Research Blood Components (Boston, MA). All samples lacked personal health information and were therefore de-identified.

### Animal ethics statement

Animal experiments were conducted in accordance with recommendations cited in the Guide for the Care and Use of Laboratory Animals of the National Institute of Health. Protocols were approved by the Laboratory Animal Care and Use Committee of Southern Illinois University School of Medicine.

### Materials

Lipopolysaccharide (LPS) derived from *Escherichia coli 055*:B5, TRI-Reagent and endotoxin-free sterile saline were from Sigma-Aldrich (St. Louis, MO). Dulbecco’s modified Eagle’s medium (DMEM), Dulbecco’s phosphate buffered saline (DPBS), and standard medium supplements were from Lonza (Basel, Switzerland). Colony stimulating factor 1 (CSF1) was from Biolegend (San Diego, CA). Mouse anesthetic cocktail consisted of ketamine (Fort Dodge Animal Health, Fort Dodge, Iowa), xylazine (Phoenix Scientific Inc., St. Joseph, MO) and sterile water. Nab-paclitaxel (nab-PXL) was purchased from a local pharmacy (Springfield, IL).

### Antibodies

The following primary anti-human antibodies were used: sheep anti-podoplanin, goat anti- VEGFR-3, anti-LYVE-1, anti-PROX1, anti-ITGA9, anti-VEGFR-2, anti-NRP2, anti-podocalyxin and anti-GFP, all from R&D Systems, Minneapolis, MN. Mouse anti-human CD14 and anti-CD68 antibodies were from Santa Cruz Biotechnology, Dallas, TX and Thermo Fisher, Waltham, MA, respectively. Rabbit anti-acetylated histone H3 and anti-Ki-67 were from Upstate, Billerica, MA and Cell Signaling Technologies, Danvers, MA, respectively. Rabbit anti-mouse Lyve-1 and rat anti-Meca-32 antibodies were from AngioBio, Del Mar, CA, and BioXCell, West Lebanon, NH, respectively. Secondary antibodies conjugated to FITC, Cy3, DyLight 488, DyLight 549, and APC donkey anti-rabbit, anti-sheep, anti-rat, anti-mouse and anti-goat IgG were all from Jackson ImmunoResearch Laboratories (West Grove, PA).

### Isolation of human monocytes from peripheral blood and mouse myeloid cells from bone marrow

#### Human cells

Human CD14^+^ monocytes were isolated from whole blood of healthy volunteers using standard methods. Briefly, blood was diluted 1:2 with DPBS supplemented with 2% FBS, layered on top of an equal volume of Lymphoprep in SepMate tubes (StemCell Technologies; Vancouver, BC), and centrifuged at 1,200 rpm for 1 hr at room temperature. Monocytes were isolated from the recovered mononuclear cells using anti-CD14 IgG-conjugated magnetic beads (Miltenyi Biotec; Cambridge, MA).

#### Mouse cells

Myeloid cells were isolated from unfractionated bone marrow (BM) of CB-17/SCID (Taconic, Hudson NY), C57BL/6 (Envigo, Indianapolis, IN) or GFP transgenic [C57BL/6-Tg(UBC-GFP)30SchaJ] mice by positive selection using anti-CD11b IgG-conjugated magnetic beads.

### Lymphatic reprogramming of CD14^+^ human monocytes and mouse bone marrow myeloid cells

Lymphatic reprogramming of human CD14^+^ monocytes and mouse CD11b^+^ BM cells was performed under identical conditions except for the use of species-specific CSF1 and time of preconditioning with CSF1 (7 days mouse or 10 days human). Cells were cultured in 6-well plates at a density of 3x10^6^ cells per well in DMEM supplemented with 10% FBS and 50 ng/ml of either human or mouse CSF1. Medium was refreshed on day 4. Following CSF1 pretreatment, cells were cultured with LPS (50 ng/ml), HMGB1 (50 ng/ml), nab-PXL (30 nM) or an equivalent volume of medium containing 50 ng/ml of CSF1 (control) for 96 hr, or pre-treated for 1hr with NF-κB inhibitors isohelenin (10μM), PDTC (5μM), or leptomycin-B (10μM) and then cultured with TLR4 ligands for 96 hrs. Differentiated cells were analyzed by RT-qPCR, flow cytometry or injected into mice pretreated with peritonitis or bearing syngeneic or xenograft breast tumors for assessment of the lymphangiogenic potential *in vivo*.

### RT-qPCR analysis and primer design

Total RNA was extracted using TRI-Reagent (Sigma Aldrich, St. Louis, MO) and 2 micrograms reverse transcribed using a RevertAid First Strand cDNA synthesis kit or SuperScript VILO cDNA Synthesis Kit (both from Thermo Fisher Scientific, Waltham, MA) according to the manufacturer’s protocols. Primers were designed to create an in-house RT-qPCR gene array specific for human inflammatory and lymphatic genes. This array contains a total of 180 targets, including 50 cytokines, 43 cytokine receptors, 40 transcription factors, 7 stem cell-specific targets, 18 endothelial-specific markers and 22 inflammatory genes. An accession number based on coding sequences was identified for each gene using NCBI database (Bethesda, MD). Accession numbers were used to obtain potential primer sequences via the Harvard Primerbank (https://pga.mgh.harvard.edu/primerbank/) or NIH qPrimerDepot (http://primerdepot.nci.nih.gov/). The sequence of each primer was compared against all known nucleotide sequences in the human genome using the NCBI BLAST alignment tool (http://blast.ncbi.nlm.nih.gov/Blast.cgi). Only primers that had unique specificity to the individual target gene were used for validation. Primers were purchased from Integrated DNA Technology (Coralville, IA). Each primer pair at a concentration of 0.5 μM was validated in duplicate using four dilutions (1, 1:10, 1:100, and 1:1000) of human universal cDNA (1 μg per reaction) prepared in-house. RT-qPCR was performed with GoTaq Green Master Mix (Promega, WI) on a MasterCycle realplex PCR machine (Eppendorf, NY). The quality of each primer was confirmed by demonstration of a single peak on melt curve analysis and amplification efficiency by Ct slope regression analysis for the four cDNA dilutions where R^2^≥0.95 was deemed acceptable. Primer sequences in S1 (human) and S2 (mouse) Tables.

A typical RT-qPCR reaction consisted of an initial denaturation step at 95°C for 1 minute followed by 40 cycles of denaturation at 95°C for 15 seconds, and annealing, extension, and data acquisition at 60°C for 1 minute. A final melt curve for each primer was calculated by heating from 60°C to 90°C. Data were normalized to β-actin and relative mRNA expression was determined using the ΔΔCt method described previously [36]. Heat-maps of the average fold-change compared to CSF1-treated myeloid cells were generated using Gene-E software from Broad Institute (Cambridge, MA).

### Flow cytometry analyses

#### Assessment of endothelial cell functions

Mouse BM CD11b^+^ myeloid cells were analyzed at three time points: 1) on day of isolation designated as *ex vivo*, 2) on day 6 of CSF1 pretreatment; and 3) on day 10 after 4 days of treatment with 50 ng/ml of LPS. Cells were analyzed for the ability to uptake FITC-labeled acetylated-low density lipoprotein (AC-LDL) (Thermo Fisher) and bind Tomato and Ulex Europaeus Agglutinin 1 (UEA1) lectins (Vector Labs, Orton Southgate, U.K.). Cells were incubated for 4 hrs at 37^°^C with complete medium containing 10μg/ml of AC-LDL or lectins, washed twice with DPBS and either imaged using an Olympus BX41 microscope (Olympus, Center Valley, PA) or fixed with 1% paraformaldehyde and analyzed with an AccuriC6 flow cytometer (BD Biosciences) to quantify number of FITC-labeled cells and mean fluorescent intensity (MFI) using FlowJo software (Tree Star, OR). Experiments were performed in duplicate and reproduced twice.

#### Expression of lymphatic markers

Human CD14^+^ monocytes were evaluated for expression of VEGFR-3, LYVE-1 and PDPN at time of isolation (ex vivo), after 10 days of treatment with CSF1, and on day 14 after being grown with TLR4 ligands for 4 days. Monocytes were first incubated with Fc receptor blocking solution (BD Biosciences, San Jose, CA) followed by incubation with 2 μg/ml of anti-VEGFR-3, -LYVE-1 and -PDPN antibodies for 30 minutes on ice before they were washed with ice-cold F-Buffer (DPBS containing 2.5% horse serum and 0.02% sodium azide) and incubated with secondary antibodies conjugated to Dylight 488- or 650. Cells from at least three healthy donors were used for each experiment with replicates performed in duplicate or triplicate. Data were acquired with an AccuriC6 flow cytometer (BD Biosciences) and analyzed with FlowJo software (Tree Star, OR). Results are expressed as mean percentage (%) of cells positive for marker expression ± standard error mean (SEM) or when applicable, mean fluorescent intensity (MFI) ± SEM. Cells treated with CSF1 alone in the absence of TLR4 ligands served as controls.

### Generation of chimera mice and LPS-induced peritonitis

Chimera C57BL/6 mice were generated by lethal irradiation with 1100cGy using a Radsource RS2000 irradiator (Brentwood, TN) followed by intravenous (i.v.) injection of 10x10^6^ unfractionated BM cells collected from the femurs of GFP^+^ transgenic mice. All mice that survived 30 days post-transplantation were considered to have full reconstitution of BM. Mice that met this criterion were injected intraperitoneally (i.p.) with 50 μg of LPS in 500 μl sterile endotoxin-free saline for 5 consecutive days, then rested for 2 days before receiving another 5-day treatment. Control mice were treated identically but injected with 500 μl sterile endotoxin-free saline only.

### Culture of mouse and human breast carcinoma cell lines

A murine breast cancer cell line generated from a transgenic C57BL/6 MMTV-PyMT model was a generous gift from Dr. DeNardo (Washington University, St. Louis, MO). Mouse breast carcinoma EMT-6 cells were purchased from ATCC (Manassas, VA) and modified in-house for expression of Firefly luciferase (EMT6-Luc). Human breast carcinoma ZR-75 cells were purchased from ATCC and modified for expression of Renilla luciferase (ZR-75-Rluc). Cells were routinely tested for mycoplasma using immunodetection kit (R&D Systems, MN). EMT6-Luc and MMTV-PyMT were screened by Impact III testing through RADIL for mouse pathogens and determined to be negative. Cell lines were cultured in DMEM containing 5% FBS, 2 mM of glutamine, 1 mM of sodium pyruvate, and 1 mM of nonessential amino acids at 37°C in 10% CO_2_. Cells were passaged twice a week by incubating for five minutes at 37°C in 0.5 mM of EDTA dissolved in DPBS followed by 0.25% of trypsin.

### Syngeneic and xenograft breast tumor mouse models

Growth of orthotopically implanted breast tumor lines was described previously [37,38]. Briefly, 0.5x10^6^ of MMTV-PyMT, 1x10^6^ of EMT6-Luc, or 4x10^6^ ZR-75-RLuc cells suspended in 50% Matrigel were implanted into the mammary fat pad (MFP) of 5–6 week-old female C57BL/6 mice, BALB/c mice (Envigo, Indianapolis, IN) or CB-17/SCID mice (Taconic, Hudson, NY), respectively. Perpendicular tumor diameters were measured 2–3 times per week using digital calipers. Tumor volume was calculated according to the formula: volume = Dd^2^π/6, where D and d are larger and smaller diameters, respectively.

### Adoptive transfer of native CD11b^+^/Pdpn^+^ and CD11b^+^/Pdpn^-^ BM subpopulations to tumor-bearing mice

CD11b^+^ BM cells isolated from GFP^+^ transgenic mice were reacted with antibodies for mouse podoplanin (Pdpn) and FACS-sorted for positive and negative fractions using the Becton-Dickinson FACSAria II high-speed cell sorter. CD11b^+^/Pdpn^+^ and CD11b^+^/Pdpn^-^ cells (2x10^6^ for each fraction) were injected i.v. into mice bearing orthotopic MMTV-PyMT or EMT6-Luc tumors (n = 5 or 6 per group) implanted 3 days prior to injection of CD11b^+^/Pdpn^-^ or CD11b^+^/Pdpn^+^ cells. Tumors were harvested at the volume of 1800 mm^3^ followed by immunofluorescent analyses for recruitment of GFP^+^ cells and density of tumor-associated lymphatic vessels.

### Adoptive transfer of *in vitro* differentiated M-LECP into LPS-treated or tumor-bearing mice

Peritonitis was induced by intraperitoneal (i.p.) injections of LPS given daily two days prior to i.v. injection of 3-5x10^6^ of *in vitro* LPS-differentiated CD11b^+^ cells derived from BM of GFP^+^ mice. Experimental M-LECP were also injected into C57BL/6, BALB/c, or CB-17/SCID mice bearing orthotopic MMTV-PyMT, EMT6-Luc, or ZR-75-RLuc tumors, respectively. On days 14, 21 and 50 after M-LECP injection, mice were anesthetized by a ketamine/xylazine cocktail and perfused with 5 mM CaCl_2_ solution. Diaphragms or tumors were collected, snap-frozen and used for determining recruitment of GFP^+^ cells, vascular integration and induction of new lymphatic vessels. Lymph nodes and lungs were collected from mice bearing ZR-75-Rluc tumors for analysis of metastatic spread.

### Determination of metastatic burden

Tissues were homogenized in ice-cold lysis buffer (Promega) containing protease inhibitors. Renilla luciferase substrate (Biosynth, Staad, Switzerland) was mixed with lysates followed by luminescence detection using a luminometer (Berthold, Oak Ridge, TN). Extracts with luciferase activity of 1,000 relative light units (RLU)/s above background were considered positive for metastasis. Data are expressed as the mean RLU/s ±SEM from duplicate readings normalized per mg of total protein determined by Bradford assay. Statistical differences between groups was determined by a Mann-Whitney test.

### Immunofluorescent staining and analysis

All antibodies were diluted 1:100 in PBST (pH 7.4, 0.1% Tween-20) containing 5 μg/ml of bovine serum albumin (BSA). Frozen sections were fixed with acetone for 10 minutes, rehydrated in PBST for 10 minutes and incubated for 1 hr at 37°C with primary antibodies. Slides were washed and incubated for 1 hr at 37°C with DyLight 488- or 549-conjugated secondary antibodies. Slides were mounted in Vectashield medium containing 4,6'-diamidino-2-phenylindole (DAPI) nuclear stain (Vector Labs, Orton Southgate, U.K.). Tumor recruitment of GFP^+^ M-LECP was analyzed by determining the number of GFP-derived pixels per field from four separate fields and normalized by total pixels. Tumor lymphatic vessels identified by anti-Lyve-1 antibody were counted and the number was normalized per section area. Fifty blood or lymphatic vessels per tumor section were assessed for co-staining with anti-GFP antibody, and the data are expressed as the percentage of vessels co-expressing GFP. Images were acquired using an Olympus BX41 upright microscope equipped with a DP70 digital camera and DP Controller software (Olympus, Center Valley, PA) at magnification indicated in figure legends.

### Statistical analysis

All results are expressed as the mean ± SEM and statistical differences were assessed by unpaired Student’s *t*-test. P values < 0.05 were considered significant.

## Results

### Activation of TLR4 expressed in human CD14^+^ monocytes upregulates lymphatic-specific genes

We previously showed that LPS activation of TLR4 caused pro-lymphatic reprogramming of an immortalized mouse macrophage line RAW264.7 [22]. The goal of this study was to determine whether primary human and mouse myeloid cells could be similarly reprogrammed by LPS or other TLR4 ligands. CD14^+^ monocytes isolated from blood of healthy donors were pretreated with CSF1 for 10 days followed by treatment with LPS, HMGB1 or nab-PXL for 4 days. These ligands represent pathogen-derived [39], host-endogenous [40], and iatrogenic [41] TLR4 activators, respectively. After 4 days of differentiation, transcripts from TLR4-activated cells were compared with those from cells treated by CSF1 alone using RT-qPCR and an in-house constructed array of genes commonly expressed in endothelial, stem/progenitor and activated immune cells (S3 Table; GEO datasets GSE75518 & GSE78162).

Table 1 shows that compared with CSF1 alone, all three TLR4 ligands induced profound shifts in human monocytes towards the vascular phenotype as evidenced by considerable upregulation of endothelial-specific genes such as CD105 (199- to 860-fold), E2F1 (75- to 200-fold), and VEGF-A (46- to 81-fold). Markers of blood vascular endothelial cells (BEC) were upregulated along with LEC-specific genes; however, the latter were upregulated at much higher levels. For instance, VEGFR-3 was upregulated up to 142-fold whereas VEGFR-1/-2, more common to BEC, were upregulated 2- to 16-fold. LYVE-1 and PDPN, specific LEC markers, increased by 28-fold and 899-fold, respectively, and expression of VEGF-C and ITGA9 was elevated 400-fold. (Table 1, LPS treatment). This was in sharp contrast to PECAM1 and TIE-2 that increased only 2 to 4-fold relative to CSF1 control.

**Table 1:** TLR4 activation of monocytes preferentially induces lymphatic differentiation.

Category	Gene	LPS	HMGB1	Nab-PXL
**Vascular-specific genes**	**CD105**	867.61 ± 29.52	662.01 ± 20.17	199.60 ± 7.16
	**E2F1**	210.88 ± 4.20	156.52 ± 2.27	75.48 ± 3.20
	**VEGF-A**	81.80 ± 2.36	73.53 ± 1.54	46.71 ± 2.52
	**NRP1**	76.49 ± 2.00	32.38 ± 0.85	9.70 ± 0.28
	**ANG2**	15.18 ± 0.40	11.42 ± 0.26	5.22 ± 0.06
	**ANG1**	34.63 ± 0.97	12.86 ± 0.46	4.32 ± 0.18
	**CD34**	6.77 ± 0.32	5.82 ± 0.26	4.03 ± 0.08
	**NRP2**	5.17 ± 0.10	1.89 ± 0.05	2.66 ± 0.05
	** **			
**Blood vascular- specific genes**	**VEGF-B**	13.72 ± 1.07	9.58 ± 0.13	4.41 ± 0.16
	**VEGFR-2**	16.08 ± 0.31	5.17 ± 0.07	2.53 ± 0.10
	**PECAM1**	2.88 ± 0.11	4.64 ± 0.16	2.09 ± 0.14
	**VEGFR-1**	7.45 ± 0.14	2.78 ± 0.08	1.93 ± 0.08
	**ETS1**	2.57 ± 0.04	1.36 ± 0.01	1.64 ± 0.06
	**HIF2A**	6.40 ± 0.14	4.46 ± 0.08	1.49 ± 0.02
	**HIF1A**	3.40 ± 0.04	2.44 ± 0.08	1.31 ± 0.03
	**TIE2**	1.92 ± 0.08	1.15 ± 0.07	1.12 ± 0.01
				
**Lymphatic-specific genes**	**PDPN**	899.78 ± 10.27	508.72 ± 15.66	225.02 ± 6.05
	**VEGF-C**	410.49 ± 16.44	194.09 ± 5.34	129.50 ± 1.42
	**ITGA9**	407.69 ± 16.84	301.86 ± 11.18	94.45 ± 4.15
	**VEGFR-3**	142.42 ± 4.36	115.38 ± 2.23	73.69 ± 1.07
	**LYVE-1**	28.78 ± 0.75	26.49 ± 0.81	15.29 ± 0.55
	**SOX18**	20.59 ± 0.45	16.80 ± 0.47	9.81 ± 0.34
	**COUPTF2**	6.68 ± 0.17	3.23 ± 0.02	5.06 ± 0.11
	**FOXC2**	8.12 ± 0.25	6.84 ± 0.13	4.53 ± 0.12
	**VEGF-D**	13.28 ± 0.42	2.96 ± 0.12	2.46 ± 0.06
	**PROX1**	3.11 ± 0.14	2.66 ± 0.08	2.44 ± 0.06

Human CD14^+^ monocytes were isolated from blood of healthy donors and cultured for 10 days with CSF1 followed by 4 days with TLR4 ligands. Samples were analyzed in triplicate and results are reported as mean fold-changes ± S.E.M. in target expression after treatment with TLR4 ligands relative to CSF1 controls. Order of the targets is based on highest response induced by nab-PXL.

**Fig 1:**
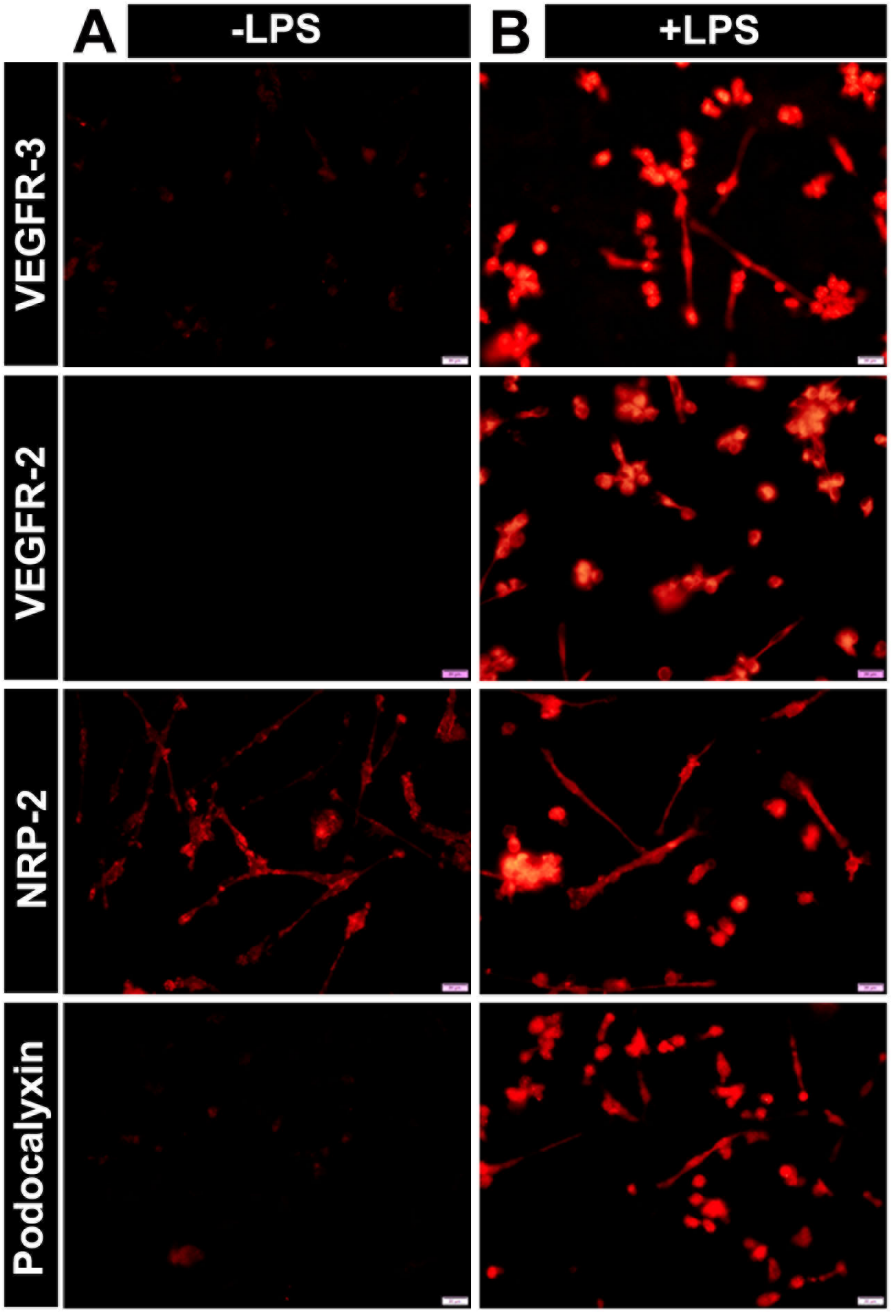
TLR4-induced myeloid-lymphatic transition is preceded by shift to vascular phenotype. Human monocytes cultured for 10 days with CSF1-supplemented medium were seeded on slides and treated with (A) growth medium with CSF1 (labeled -LPS) or (B) medium containing CSF1 and 50 ng/ml of LPS (labeled +LPS). After 4 days of treatment, cells were stained for vascular endothelial markers VEGFR-3, VEGFR-2, NRP-2, and podocalyxin. All images were acquired at 400X magnification.

Immunohistochemical analysis confirmed the overall pro-vascular shift of monocytes treated for 10 days with CSF1. As shown in Fig 1, cells treated with LPS (but not those treated with CSF1 alone) expressed VEGFR-3, VEGFR-2, neuropilin-2 (NRP2), and podocalyxin, all of which are expressed in both BECs and LECs [42].

Comparison of pro-vascular phenotype at different timepoints of differentiation revealed that some endothelial-specific properties are induced by CSF1 alone and further increased upon TLR4 activation. For instance, CSF1 treated cells demonstrated abilities to bind acetylated LDL (AC-LDL), UEA1 and Tomato lectins (S1 Fig), properties that are typically attributed only to endothelial cells [43,44]. However, following this treatment lectin binding nearly doubled whereas AC-LDL uptake increased by >30%. At the end of LPS differentiation, 100% of cells exhibit these endothelial functions (S2 Fig) with the efficiency surpassing that of freshly isolated cells by 123-fold, 17.6-fold and 8.64-fold for binding of AC-LDL, UEA1 and Tomato lectins, respectively (S1 Fig).

We also noted that CSF1 treatment alone significantly increased division of attached cells at days 4–6. This was evident by increase in cells with double nuclei identified by anti-acetylated histone H3 antibody and positivity for Ki-67 (S3 Fig). Cell numbers continued to increase up to day 10 in culture but plateaued when exposed to differentiation agents (not shown). This is consistent with independent reports on proliferation of monocyte-derived progenitors cultured in CSF1-containing medium [45].

**Fig 2:**
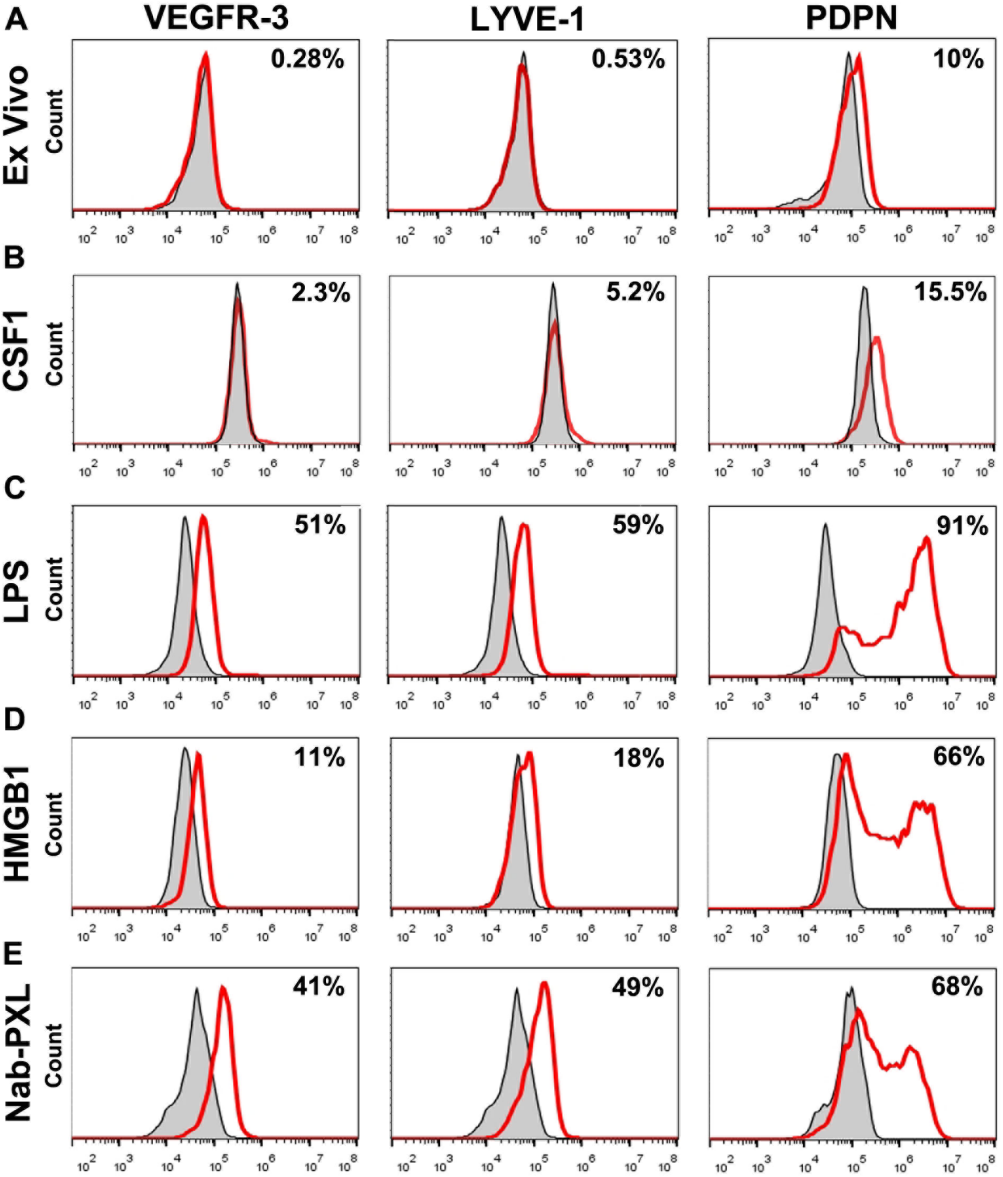
TLR4-mediated activation of human monocytes induces a lymphatic endothelial phenotype. CD14^+^ monocytes were isolated from the blood of healthy donors using anti-CD14 IgG conjugated magnetic beads. (A) Overlays of representative flow cytometry histograms demonstrating expression of lymphatic markers VEGFR-3, LYVE-1 and PDPN for freshly isolated cells stained with marker-specific antibodies (red line) or isotype controls (grey area). (B, C, D, E) Cells were cultured for 10 days with human CSF1 followed by 4 days with CSF1-supplemented medium with or without TLR4 ligands. Overlays of representative histograms demonstrate expression of VEGFR-3, LYVE-1 and PDPN in cells treated with CSF1 only (B) or CSF1 and (C) LPS (50 ng/mL), (D) HMGB1 (50 ng/mL), or (E) nab-PXL (30 nM). All analyses were performed in duplicate and reproduced using monocytes from three different donors. Representative histograms for each target, time point, and differentiation stimulus are shown.

Flow cytometry analysis showed that monocytes activated by three different TLR4 ligands upregulate VEGFR-3 and other LEC-specific proteins, LYVE-1 and podoplanin (PDPN) (Fig 2). CSF1 alone (10 days treatment) increased the percentage of positive cells from <1% in ex-vivo monocytes to 2–5%. In contrast, treatment with LPS or nab-PXL increased this fraction to 50–60% with more modest response for HMGB1 (10–18%). PDPN was present in a small fraction (6–10%) of freshly-isolated monocytes prior to differentiation. However, the number of PDPN^+^ cells was significantly increased after treatment with LPS, HMGB1 or nab-PXL rising to 91%, 66%, and 68% of monocytes, respectively (Fig 2).

**Fig 3:**
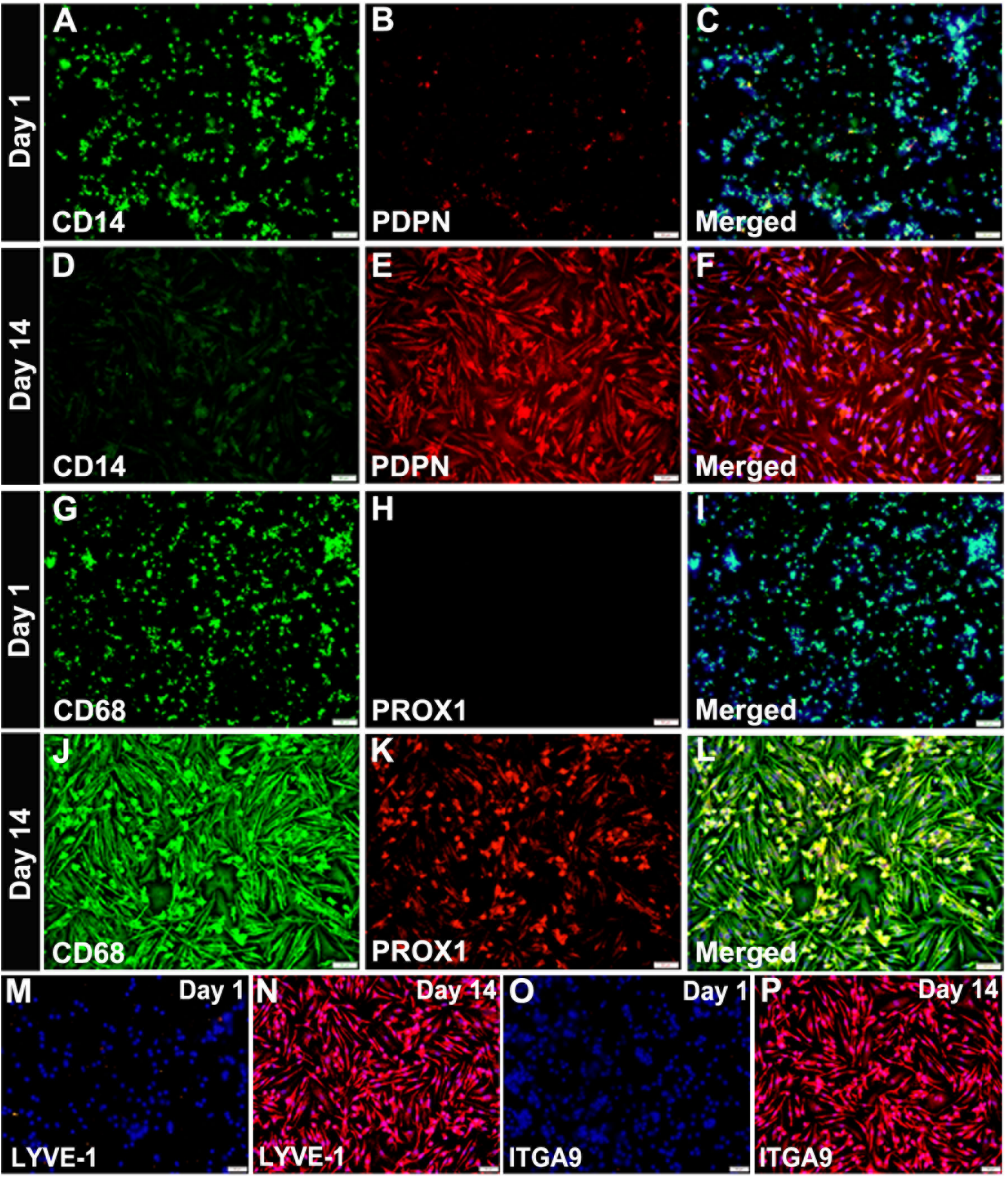
TLR4-reprogrammed monocytes upregulate LEC-specific proteins and downregulate some myeloid markers. CD14^+^ monocytes seeded on slides were double-stained on Day 1 in culture or on Day 14 (10 days with CSF1 followed by 4 days LPS) for expression of myeloid markers (A, D) CD14, and (G, J) CD68, and lymphatic-specific proteins (B, E) PDPN, and (H, K) PROX1. Double-staining of myeloid and LEC markers (A to L) used FITC- and Cy3-conjugated secondary antibodies, respectively. Single staining with Cy3-conjugated secondary antibodies was performed on cells at day 1 and 14 to detect LYVE-1 (M, N) and ITGA9 (O, P). Merged images of myeloid and lymphatic proteins detected in the same cells are shown in panels C, F, I and L. All images were acquired at 200X magnification.

Acquisition of the lymphatic phenotype was confirmed for proteins by flow cytometry and immunohistochemical approaches. Both methods showed that ex-vivo and cells cultured for 1 day express negligible levels of LEC markers (Figs 2 and 3). As shown in Fig 2B, CSF1 treatment alone without TLR4 stimuli increased expression of LEC markers only by 2–5%. In contrast, all tested LEC markers were highly upregulated on day 14 (Figs 2, 3E, 3K, 3N and 3P). CD14, a myeloid marker, was clearly present on day 1 but absent on the last day of differentiation (Fig 3D). However, some myeloid markers persisted and even increased by day 14 (e.g., CD68, Fig 3G and 3J). Thus, acquisition of new lymphatic markers paralleled loss of some, but not all, myeloid markers resulting in the broadly reported mixed phenotype [12,24]. Collectively, these data show that TLR4 activation of human monocytes induces not only generally pro-vascular gene expression but preferential LEC differentiation as indicated by specific LEC markers on mRNA and protein levels (Table 1, Figs 1, 2 and 3).

### Myeloid-lymphatic transition (MLT) coincides with increase of inflammatory proteins

**Fig 4:**
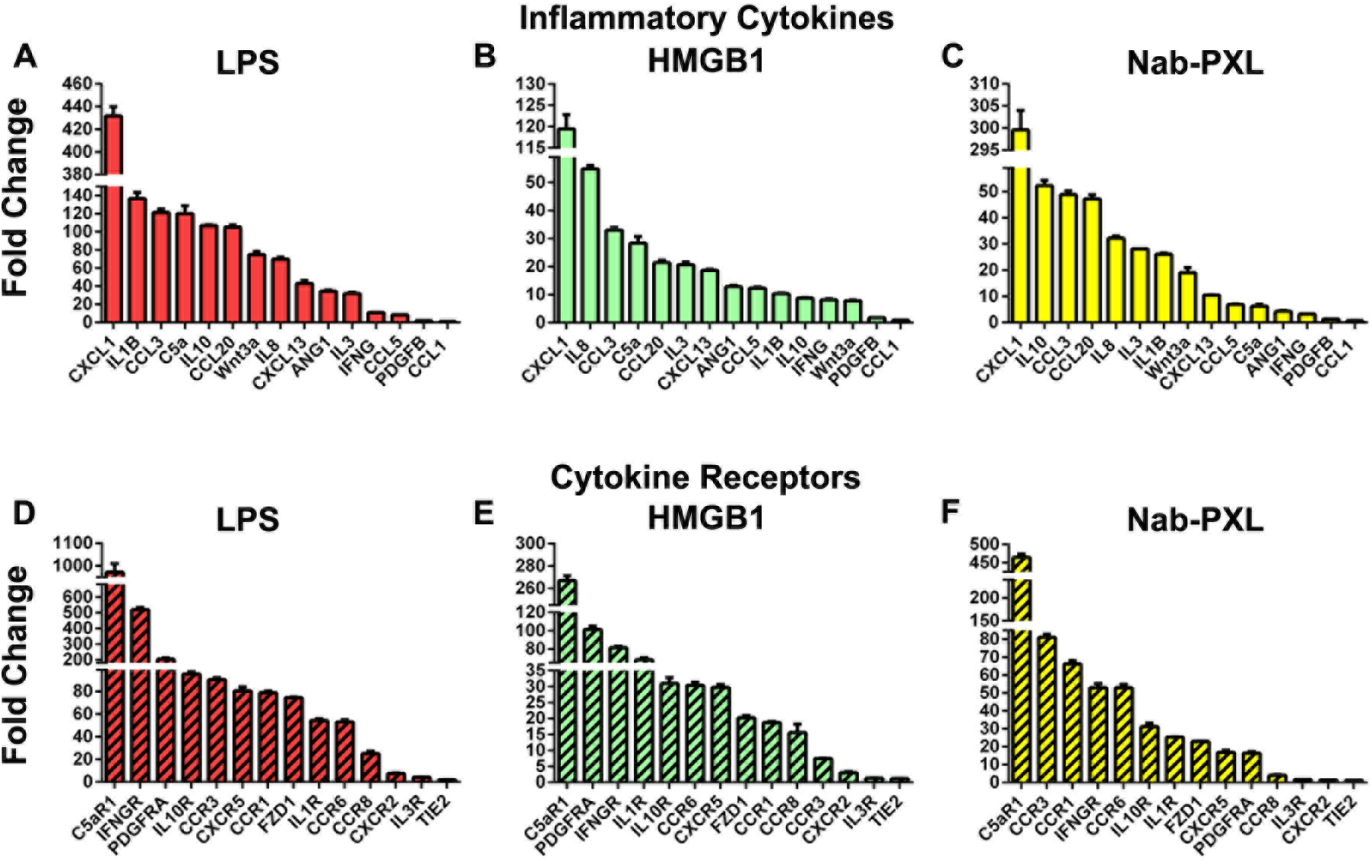
Lymphatic reprogramming of human monocytes is accompanied by upregulation of inflammatory cytokines and receptors. CD14^+^ monocytes were treated with CSF1 alone (control) or additionally differentiated with (A, D) LPS, (B, E) HMGB1 or (C, F) nab-PXL. After 4 days of treatment, expression profiles of cells in control and TLR4 ligand-treated groups were compared using PCR arrays. All experiments were reproduced twice with each target analyzed in triplicate and normalized to β-actin. Results for each target are reported as mean fold-change in treated cells compared with the control group ± S.E.M. Results for selected targets are depicted with collective data provided in S3 Table under sub-groups “Cytokines” and “Cytokine Receptors”.

**Fig 5:**
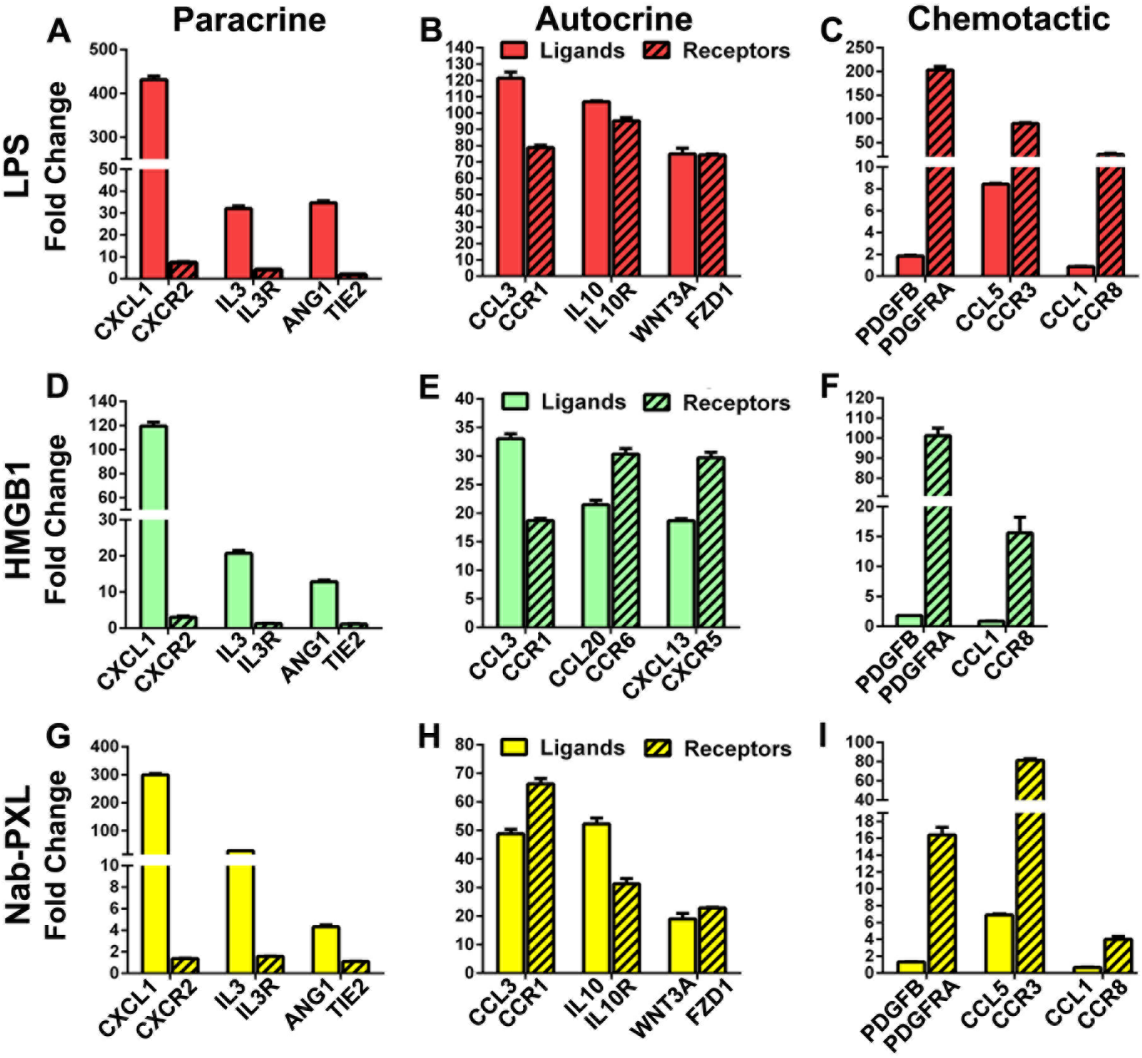
Cytokines upregulated by TLR4 signaling might play autocrine, paracrine and chemotactic roles. Human monocytes differentiated by LPS, HMGB1 and nab-PXL were analyzed by RT-qPCR as described under Methods. Matched pairs of cytokines and receptors were grouped based on expression pattern suggestive of roles in (A, D, G) paracrine, (B, E, H) autocrine, or (C, F, I) chemotactic signaling pathways. Results are plotted as mean fold-change ± S.E.M in CD14^+^ cells treated with TLR4 ligands as compared with CSF1 alone.

As expected, activation of TLR4 significantly increased a variety of inflammatory cytokines, many of which were co-upregulated with corresponding receptors (Figs 4 and 5 and S3 Table). Nearly 38% of all examined cytokines (n = 49) and corresponding receptors (n = 42) were upregulated higher than 50-fold after LPS treatment. In comparison, an analogous transcriptional shift was detected for 12% of genes after treatment with HMGB1 or nab-PXL. As shown in Fig 4A, 4B and 4C, the strongest induction by all three TLR4 ligands was detected for CXCL1 (119- to 431-fold increase), CCL20 (21- to 105-fold), and CCL3 (33- to 121-fold). Cytokines such IL-5, IL-6, IL-8, and IL-10 were highly elevated by at least two of three tested TLR4 ligands (S3 Table). All three ligands strongly upregulated C5aR1and IFNGR whereas at least 2 out of 3 ligands elevated PDGFRA, IL10R, CCR3, CCR6, FZD1, and IL1R. The least upregulated cytokines in all groups were PDGFB and CCL1 while in the category of receptors, TIE2 was relatively unchanged (Fig 4D–4F).

Analysis of the levels of upregulated cytokines and corresponding receptors suggested that this class of targets may contribute to MLT by three mechanisms: 1) paracrine stimulation of surrounding stroma cells; 2) self-activation through autocrine loops; and 3) chemotaxis of differentiated M-LECP towards the inflamed site. The paracrine role is indicated by a greater fold-increase of soluble factors compared with levels of matching receptors as shown for CXCL1-CXCR2, IL3-IL3R, and other pairs (Fig 5A, 5D and 5G). The autocrine role is suggested by comparable fold-increase for both ligands and receptors as noted for CCL3-CCR1, IL10-IL10R, and other sets (Fig 5B, 5E and 5H). A chemotactic role is suggested by substantial upregulation of receptors such as PDGFRA and CCR8 in the absence or negligible increase for matched ligands (Fig 5C, 5F and 5I). This suggests that these receptors might mediate directional migration of differentiated monocytes to tumors and chronically inflamed sites, which produce high quantities of corresponding ligands. Despite some differences in upregulated targets, multiple pairs were induced for all three stimuli of TLR4 (e.g., CXCL1-CXCR2, CCL3-CCR1, CCL1-CCR8). This overlap suggests that these proteins induced by TLR4 activation along with LEC-specific markers serve a variety of paracrine, autocrine, and chemotactic roles that might be necessary for advancing M-LECP differentiation and/or migration to the inflamed tissue.

### Activation of TLR4 in mouse BM-derived myeloid cells also induces pro-lymphatic reprogramming

While analysis of human MLT is critical for understanding pathological conditions, mouse remains the main specie for modeling human diseases. We, therefore, wished to compare TLR4-dependent transcriptional changes in human and mouse primary myeloid cells undergoing MLT. Although mouse BM-derived lymphatic progenitors have been implicated in promoting lymphatic formation [13,15], the gene expression profile of this subset has not been determined. To compare mouse and human MLT, we first determined the gene expression profile of primary BM-derived mouse myeloid cells cultured under similar conditions to human monocytes. CD11b^+^ BM-derived cells from immunodeficient and immunocompetent mice were treated with mouse CSF1 for 7 days followed by treatment with one of the TLR4 ligands, nab-PXL, for 4 days. We elected to use nab-PXL in this assay because this drug is a potent activator of TLR4 as shown here (S3 Table) and reported previously [46], and because it mediates recruitment of myeloid cells to human tumors [47] and demonstrates pro-lymphangiogenic activity in mouse tumor models [38], both of which are highly relevant to cancer patients undergoing taxane treatment. For comparative purposes, we selected 43 representative targets from >180 analyzed for human CD14^+^ cells. Transcript levels were determined by RT-qPCR using mouse-specific primers and normalized to beta actin. Results are reported as mean fold-change in each target for cells treated with CSF1 alone (control) compared with those differentiated by nab-PXL.

**Fig 6:**
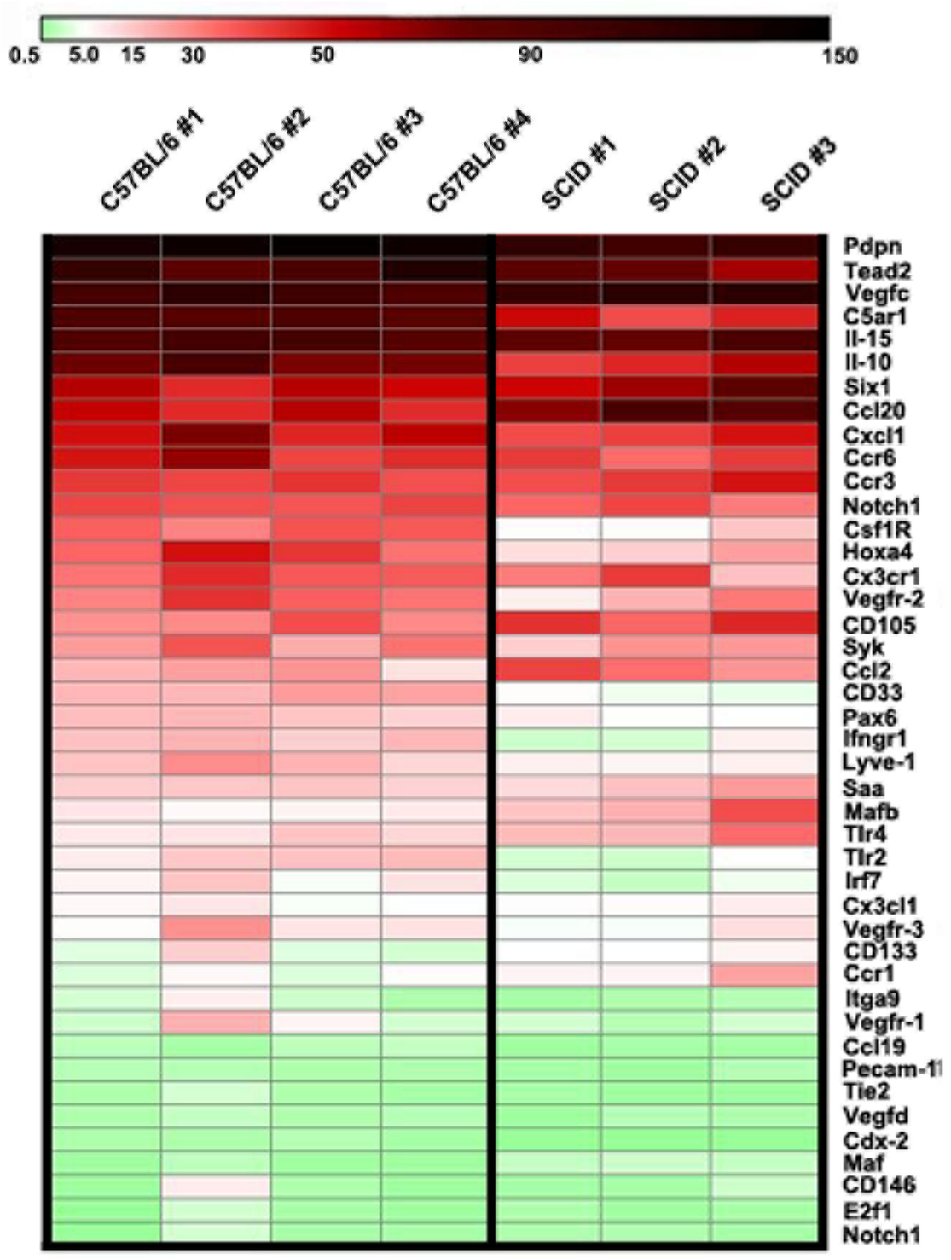
TLR4 activation induces lymphatic reprogramming of myeloid cells isolated from BM of immunocompetent and immunodeficient mice. CD11b^+^ cells isolated from BM of C57BL/6 mice (n = 4) and CB-17/SCID mice (n = 3) were used to induce M-LECP differentiation using nab-PXL (30 nM). Control cells were treated for identical time with medium containing only CSF1. After 4 days of nab-PXL or control treatment, cells were used to extract RNA for RT-qPCR analysis of representative targets (n = 43) performed in triplicate. Heat-maps of transcriptional changes detected in individual C57BL/6 and CB-17/SCID mice are shown. Beta actin-normalized data reported as mean fold-change ± S.E.M are shown in S4 and S5 Tables for C57BL/6 and CB-17/SCID mice, respectively. Rows correspond to listed genes and columns to individual mice with mouse strains indicated at the top. Scale bar denotes relative downregulation, no change, and upregulation as indicated by green, white and red colors, respectively.

Using this approach, we performed seven independent PCR analyses of BM cells from four C57BL/6 and three CB-17/SCID (BALB/c genetic background) female mice, 4–6 weeks of age. Data from individual mice were highly consistent with S.E.M. less than 10% for majority of targets (Fig 6 and S4 and S5 Tables). Heat-maps in Fig 6 show substantial overlaps in gene expression among all seven tested mice suggesting that TLR4-induced gene expression occurs independently of immune status or strain.

**Fig 7:**
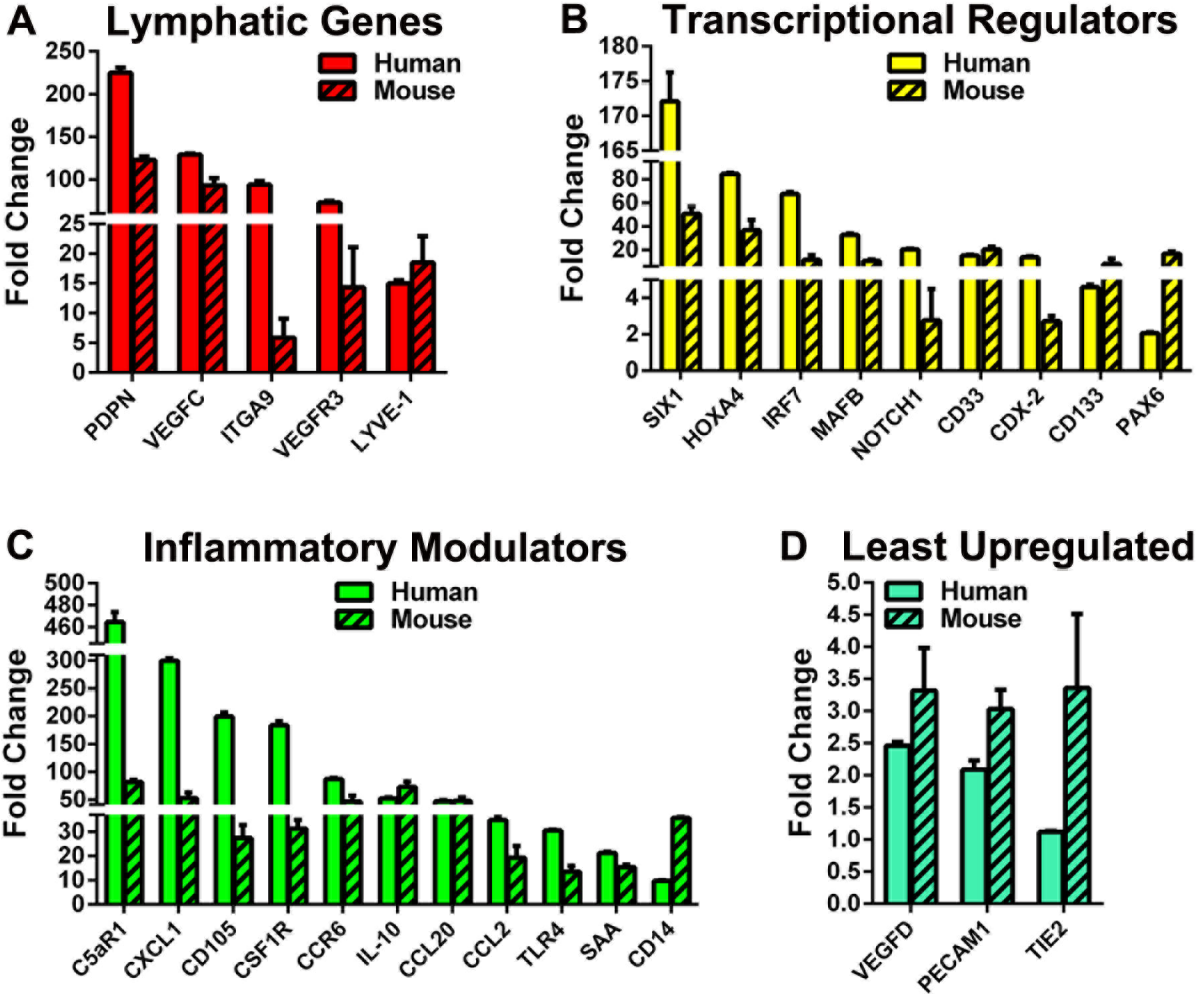
Human and mouse myeloid cells share similar patterns of TLR4-induced lymphatic reprogramming. Human and mouse cells were treated with specie-specific CSF1 followed by treatment with nab-PXL (30 nM) before RT-qPCR analysis. Data for all analyzed targets (n = 43) are shown in S3 and S4 Tables for human monocytes and mouse cells from immunocompetent C57BL/6 strain, respectively. Similarities in gene expression are shown by grouping targets based on properties such as (A) lymphatic-specific genes; (B) transcriptional regulators; (C) inflammatory modulators; and (D) relatively unchanged genes. Results are presented as mean fold change ± S.E.M. for human and mouse cells treated with nab-PXL compared with cells treated with CSF1 alone for identical period of time.

Most genes were similarly regulated in myeloid cells from immunodeficient and immunocompetent mice although occasional targets were induced to a lower level in SCID mice. Out of 43 targets, 61% were consistently upregulated (16/43) or downregulated (10/43) in both C57BL/6 and SCID mice. Moreover, the gene profile detected for mouse BM myeloid cells was similar to that of human monocytes activated by TLR4 ligands (Fig 7). Among multiple similarities, we noted high-level increases of lymphatic-specific markers (Fig 7A), stem- and progenitor-specific transcription factors (Fig 7B) and inflammatory proteins (Fig 7C). In agreement with data from human monocytes, PECAM1 and TIE2 were only slightly increased or unchanged in mouse myeloid cells (Fig 7D). Likewise, VEGF-D, previously reported to be unaffected by LPS treatment of immortalized mouse macrophages [22], was the least responsive gene to TLR4 activation in both primary human and mouse myeloid cells.

In summary, these data suggest that TLR4-dependent MLT can be generalized to human and mouse species regardless of a specific strain, and that this process is largely independent of T and B lymphocytes absent in the immunodeficient SCID mice. These data also establish for the first time the rational basis for using mouse primary BM cells and *in vivo* models to gain better understanding of the M-LECP role in human disorders.

### Myeloid-lymphatic transition (MLT) is prevented by NF-κB inhibitors

We previously demonstrated that VEGFR-3 expression is regulated by NF-κB [48], and that MLT in myeloid cells directly depends on autocrine activation of VEGF-C/VEGFR-3 signaling [22]. This led us to propose that inhibition of NF-κB not only suppresses VEGFR-3 but also other LEC genes that are subsequently regulated by VEGFR-3 signaling. This hypothesis is consistent with the well-established role of NF-κB as the main mediator of TLR4 pathway [49] as well as with early upregulation of VEGFR-3 during MLT [22]. To test this hypothesis, we induced MLT in human CD14^+^ cells using LPS, HMGB1 or nab-PXL either under standard conditions (see Methods) or in the presence of NF-κB inhibitors isohelenin [50], PDTC or leptomycin-B [51,52]. Isohelenin is highly specific inhibitor of NF-κB and the other two drugs have predominant NF-κB specificity [50–52]. Expression of LEC-specific genes PDPN, LYVE-1, VEGFR-3, VEGF-C, PROX1, and ITGA9 was determined 96 hours post-treatment.

**Fig 8:**
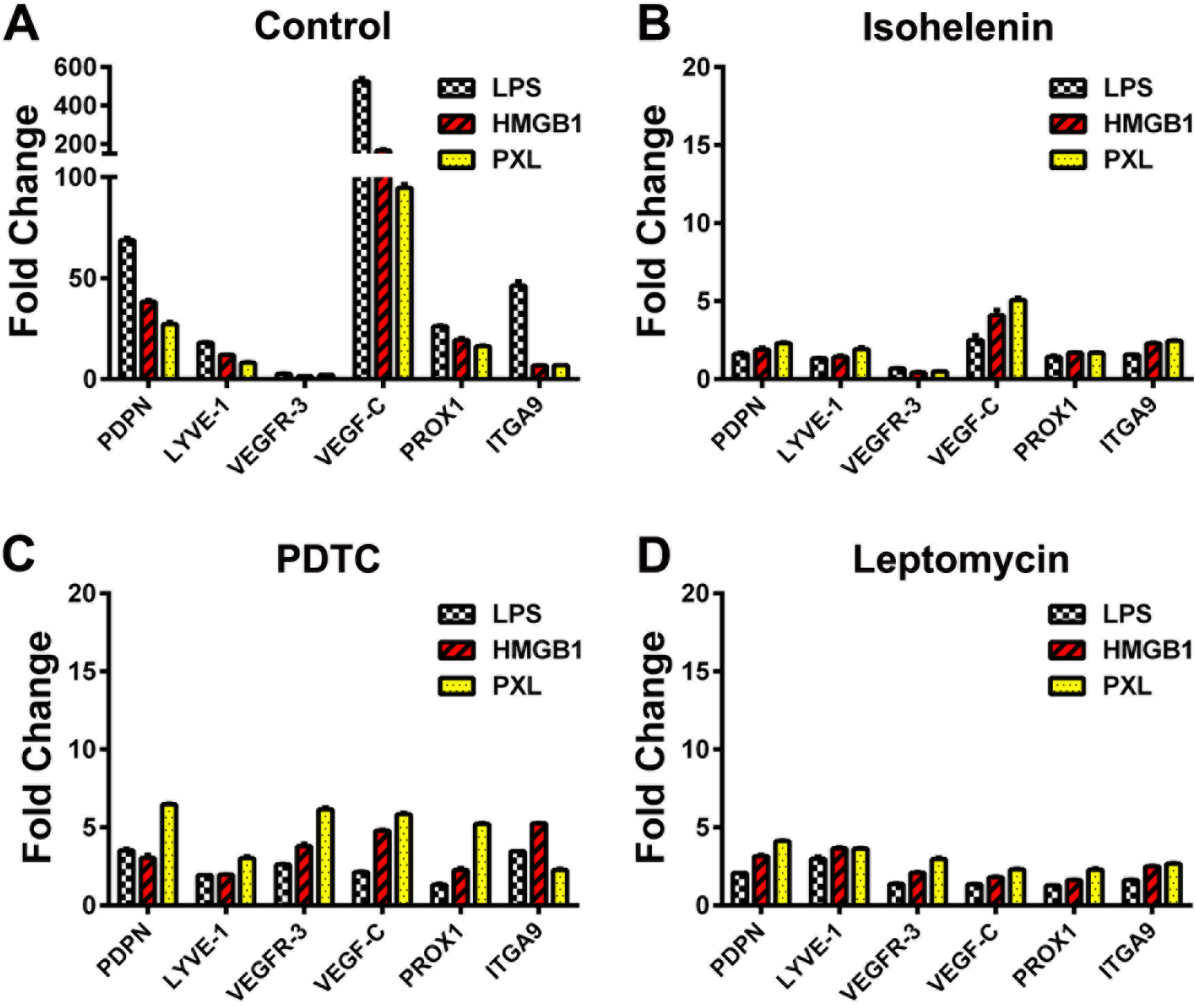
NF-κB inhibitors significantly suppress MLT. Four sets of human monocytes were differentiated with LPS, HMGB1 or nab-PXL as described under Methods. One set was treated with only TLR4 ligands and served as control (A). Three other sets were additionally treated with (B) 10 nM of isohelenin, (C) 5 μM of PDTC, or (D) 10 nM of leptomycin-B. After 4 days of treatment, mRNA was extracted and used to determine expression of the indicated lymphatic genes. The analyses for each condition and gene target were performed in triplicate. Results are presented as mean fold-changes of beta actin normalized values ± S.E.M. Note 30-fold difference in the scale of the Y-axis of control plotted data (A) and that from NF-κB inhibitors treated cells (B-D).

Fig 8 shows that all three inhibitors substantially suppressed expression of LEC genes. The most effective inhibitor was an NF-κB-restricted drug isohelenin that reduced expression of LEC transcripts to baseline levels (96–98% inhibition, Fig 8B). VEGFR-3 was the least affected gene likely because its upregulation peaks at 24 hours rather than 96 hours [22]. VEGF-C was the most affected gene with 95–99% inhibition in the presence of anti-NF-κB drugs as compared with controls (Fig 8B–8D). PDTC and leptomycin-B produced similar effects in suppressing LPS- and HMGB1-induced LEC transcripts whereas the effects on paclitaxel-induced transcription were less pronounced with 60–90% inhibition compared with controls. Collectively, these data strongly support our hypothesis that NF-κB is directly responsible for mediating TLR4 signals in myeloid cells and their reprogramming into M-LECP.

### Endogenous M-LECP substantially contribute to lymphangiogenesis *in vivo*

The lymphangiogenic activity of endogenous M-LECP has been documented in several previous studies that utilized either chimera mice reconstituted with BM from GFP-expressing mice [15] or an isolated BM-derived CD11b^+^/Pdpn^+^ fraction thought to represent M-LECP precursors [25]. We used both of these assays to determine the lymphangiogenic potential of endogenous M-LECP. In the first experiment, mice fully reconstituted with GFP^+^ BM were treated with LPS to induce peritonitis [22] followed by assessment of GFP^+^/CD11b^+^ cell recruitment to inflamed diaphragms. In the second experiment, BM-derived GFP^+^/CD11b^+^ cells from healthy mice were FACS-sorted into Pdpn-positive and Pdpn-negative fractions followed by injection into mice bearing syngeneic orthotopic breast carcinoma EMT6-Luc.

**Fig 9:**
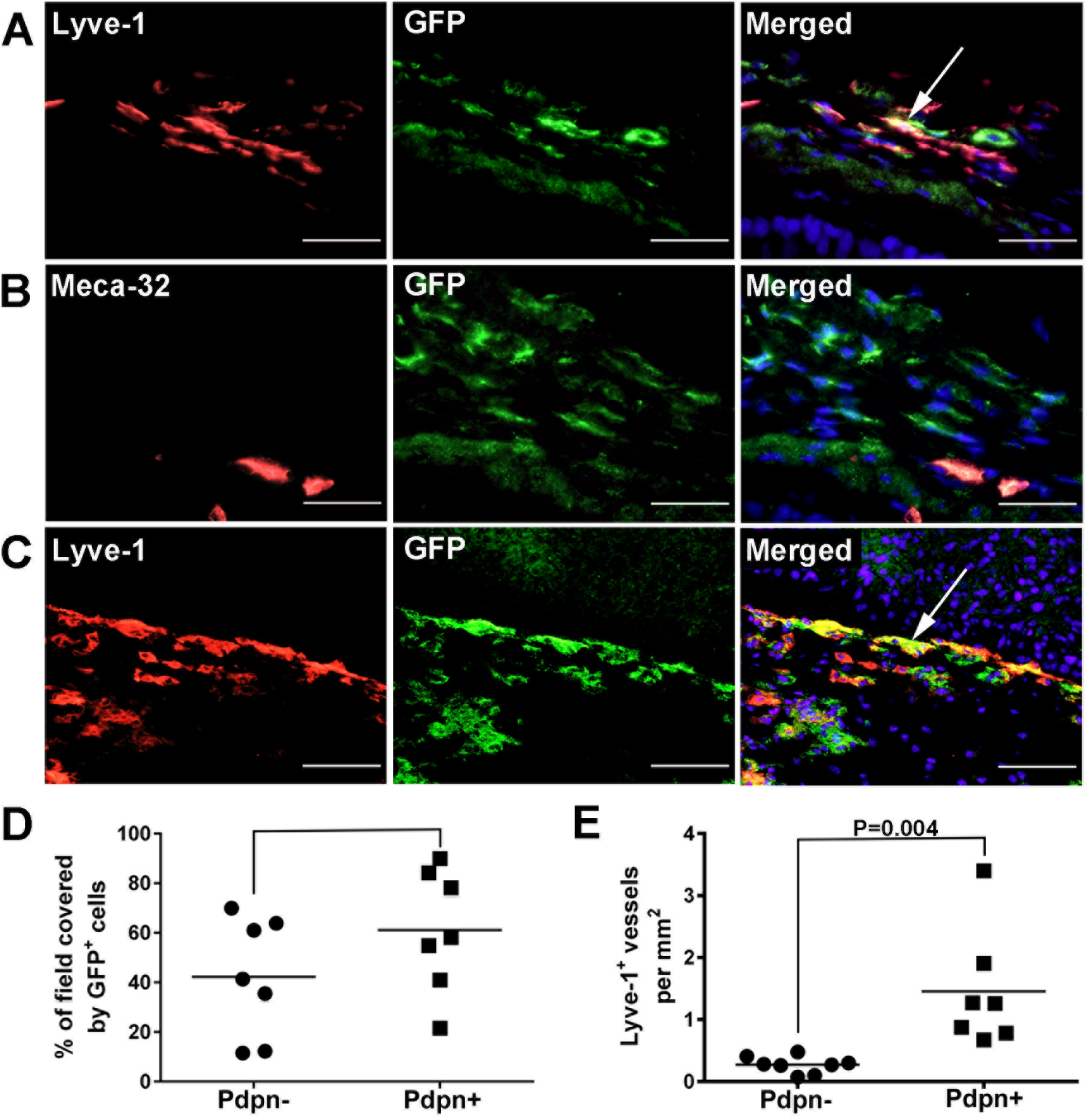
Endogenous M-LECP promote inflammatory and tumor lymphangiogenesis *in vivo*. (A, B) C57BL/6 female mice were subjected to a lethal dose of radiation and injected with BM cells from GFP transgenic mice. Once BM reconstitution was complete (4 weeks later), mice received daily injections of LPS (50 μg) of for two weeks to induce chronic peritonitis. At this time, diaphragms were removed and stained with antibodies to GFP and **(**A) Lyve-1 or (B) Meca-32. (C) EMT6-Luc tumor bearing mice were injected with 5x10^6^ of GFP^+^/CD11b^+^/Pdpn^-^ or GFP^+^/CD11b^+^/Pdpn^+^ BM cells (6–7 mice per group). EMT6-Luc tumors were harvested 14 days later and stained with antibodies to Lyve-1 and GFP. Images were acquired at 400X magnification. (D) Recruitment of CD11b^+^/Pdpn^-^ and CD11b^+^/Pdpn^+^ monocytes to EMT6-Luc tumors was quantified by determining the number of GFP^+^ pixels per field from four fields per section. (E) Peritumoral LVD of EMT6-Luc tumors was calculated by counting all peritumoral Lyve-1^+^ vessels on a section and normalizing per section area. Square and dot symbols represent Pdpn-positive and Pdpn-negative CD11b^+^ cells, respectively. Each symbol represents a value obtained from an individual mouse and reports the percentage of field covered by GFP cells or the density of Lyve-1^+^ vessels, respectively. The black bar indicates the mean for each group. Statistical significance was calculated by Student’s t-test with P-value indicated above black bracket.

Both models showed substantial recruitment of BM-derived GFP^+^ cells to the inflamed diaphragms or tumors (Fig 9A and 9C) with some preference for Pdpn-positive compared with Pdpn-negative cells (Fig 9D). In chimera mice reconstituted with unfractionated BM, some specimens showed occasional overlap between GFP and Meca-32 staining. However, in all tested models the frequency of M-LECP integration into Lyve-1^+^ lymphatic vessels (Fig 9A and 9C) was much higher than into blood vasculature (Fig 9B). Importantly, delivery of a Pdpn^+^ fraction, but not Pdpn^-^ myeloid cells, highly correlated with increased lymphatic vessel density (Fig 9E, P = 0.004) supporting previous report on the major role of CD11b^+^/Pdpn^+^ BM-derived cells in adult lymphangiogenesis [25]. This is consistent with our results showing highly upregulated podoplanin in TLR4-activated human monocytes as well as mouse myeloid cells (Figs 2, 3 and 6). This is also in line with prior observations that BM-derived LEC-positive myeloid cells are significant contributors to inflammatory [13,16,22] and tumor lymphangiogenesis [23,53].

### *In vitro* differentiated M-LECP are capable of promoting tumor lymphangiogenesis *in vivo*

**Fig 10:**
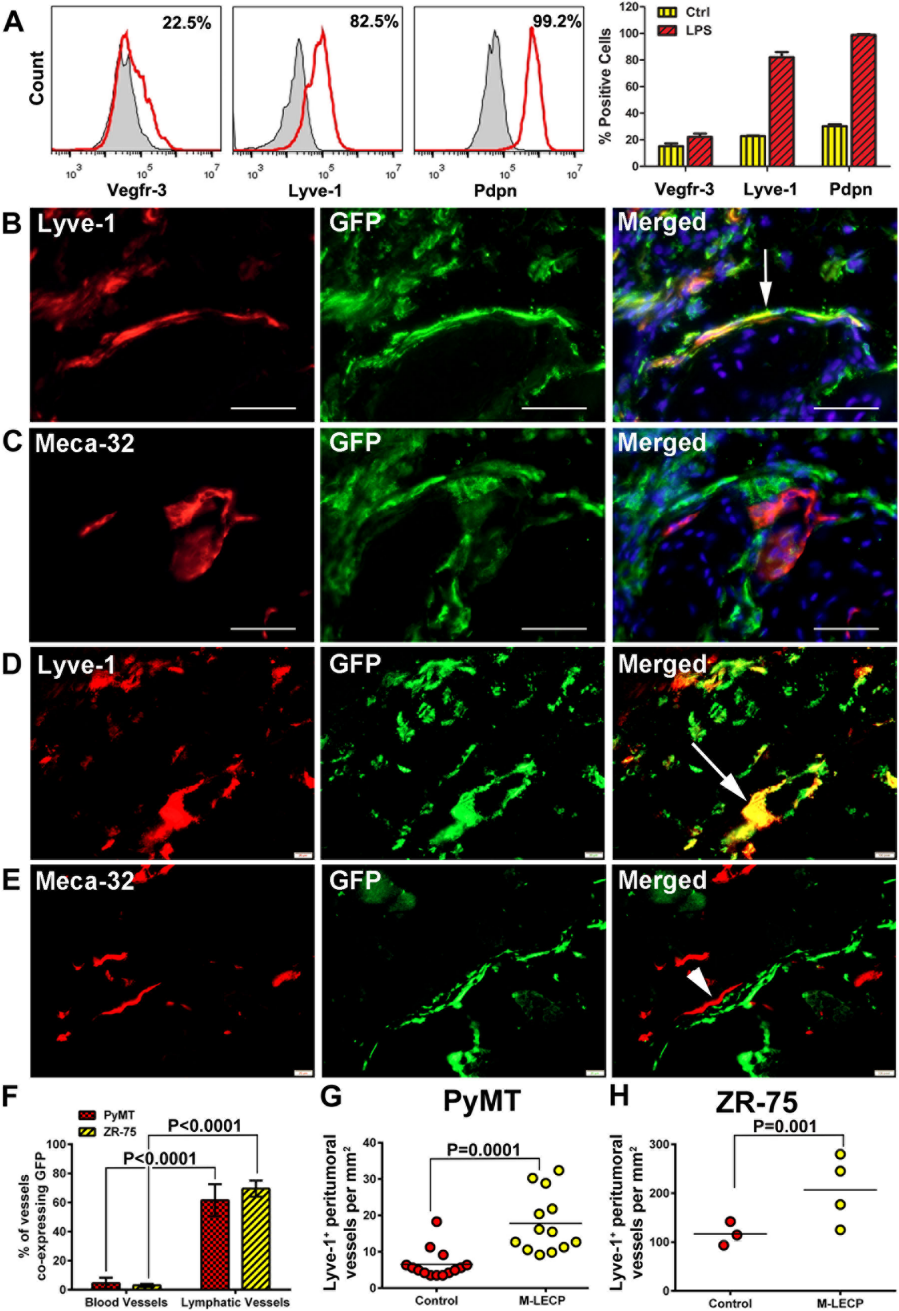
TLR4-induced M-LECP differentiated *in vitro* promote tumor lymphangiogenesis *in vivo*. CD11b^+^ cells isolated from BM of GFP transgenic mice were differentiated with LPS and injected into tumor-bearing mice. (A) Overlays of representative histograms demonstrating expression of Vegfr-3, Lyve-1 and Pdpn for cells treated with CSF1 only (control, grey area) or CSF1 plus LPS to induce lymphatic differentiation (red lines). The mean % of positive cells for LEC markers from two independent experiments ± S.E.M. are shown. (B, C) Co-localization of GFP^+^ cells with Lyve-1^+^ (B) or Meca-32^+^ (C) vessels in a syngeneic breast cancer MMTV-PyMT tumors. (D, E) Co-localization of GFP^+^ cells with Lyve-1^+^ (D) or Meca-32^+^ (E) vessels in a human xenograft breast cancer ZR-75 model. All images were acquired at 400X magnification. (F) Quantification of blood and lymphatic vessels with integrated GFP. (G, H) Changes in peritumoral LVD in mice bearing MMTV-PyMT (G) or ZR-75 (H) tumors injected with M-LECP as compared with saline-injected controls.

Next, we tested the ability of *in vitro* TLR4-differentiated M-LECP to integrate and increase the density of tumor-induced lymphatic vessels. Mouse M-LECP differentiated from CD11b^+^ BM of GFP transgenic mice were injected i.v. into mice bearing mouse EMT6-Luc, MMTV-PyMT, or human ZR-75 breast tumors. Tumors harvested 14 days after M-LECP injection were stained with antibodies to GFP, Lyve-1, or Meca-32. Fig 10A shows a typical profile of M-LECP injected into mice demonstrating that cells were highly positive for Pdpn and Lyve-1 (~100% and 87%, respectively) with modest expression for Vegfr-3 (22%). The relatively low percentage of VEGFR-3^+^ cells is consistent with transient VEGFR-3 expression peaking at 24 hours from the onset of MLT [22]. Therefore, cells harvested at 96 hours are expected to be highly positive for Lyve-1, Pdpn, and other LEC markers but express a relatively low level of VEGFR-3.

Exogenous GFP^+^ M-LECP predominantly integrated into Lyve-1^+^ lymphatic vessels as opposed to Meca-32^+^ blood vessels (Fig 10B and 10C). This conclusion was reached after quantifying integration events that were identified in 3–4% of blood vessels as opposed to 50–70% of lymphatic vessels in both analyzed tumor models (P<0.0001, Fig 10F). It should also be noted that yellow color indicating co-localization of GFP and Lyve-1 expression was often evident throughout the entire vascular structure as shown in Fig 10D. This staining pattern indicates that GFP protein is expressed in the cytosol of Lyve-1^+^ endothelial cells suggesting that GFP^+^ M-LECP donated their mRNA and/or proteins to existing LECs. Importantly, in both tumor models, the densities of peritumoral lymphatic vessels were significantly increased by 2.0- to 2.7-fold in mice injected with M-LECP compared with controls (P = 0.001; Fig 10G and 10H). This evidence from orthotopic breast cancer models demonstrates that TLR4 activation of BM myeloid cells generates functional M-LECP capable of expanding tumor lymphatic vasculature *in vivo*.

### M-LECP driven tumor lymphangiogenesis enhances breast tumor metastasis

Despite multiple reports demonstrating integration of lymphatic progenitors into preexisting lymphatic vessels [12,15,29] and their role in increasing the number of these vessels [25,32], the evidence for impact of progenitors on lymphatic function is still very limited. Specifically, neither endogenous nor exogenous M-LECP were shown to directly affect tumor growth and metastasis. We presented here new evidence showing that *in vitro* differentiated M-LECP significantly increase tumor-associated LVD (Fig 10G and 10H). This experimental setting allowed us to analyze the functional impact of M-LECP on tumor growth and metastasis.

**Fig 11:**
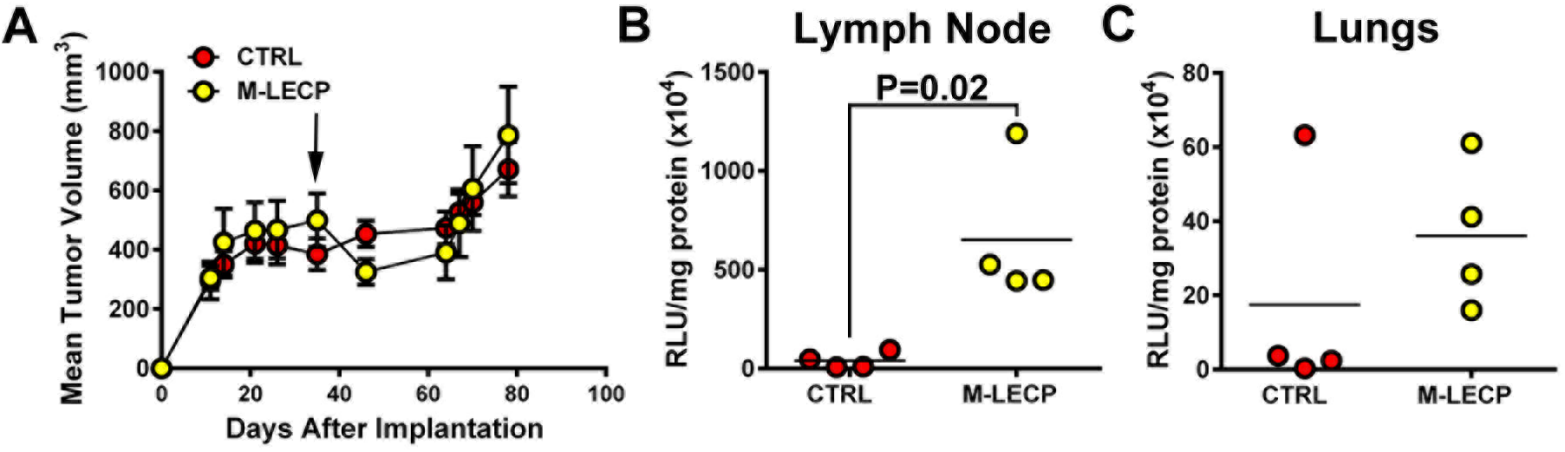
*In vitro* produced M-LECP increase lymphatic metastasis *in vivo*. Renilla luciferase-tagged ZR-75 tumor cells were orthotopically implanted into mammary fat pad of immunodeficient SCID mice and allowed to reach 500 mm^3^ before injecting mice i.v. with 2x10^6^ of *in vitro* LPS-differentiated mouse M-LECP or saline control (4 mice per group). (A) Tumor growth was monitored 2–3 times per week. Metastatic burden to lymph nodes (B) and lungs (C) was analyzed 45 days after M-LECP injection by measuring Renilla luciferase activity and normalizing per mg of total protein in tissue lysates. Each dot represents metastatic burden for an individual mouse expressed as RLU/mg of protein x 10^4^. The black bracket indicates statistically significant difference between control and M-LECP treated groups (n = 4) determined by a Mann-Whitney test with P-value listed above the line.

Injection of M-LECP into mice with well-established ZR-75 tumors (~500 mm^3^) transiently decreased tumor growth (Fig 11A) albeit the difference compared with control tumors did not reach statistical significance. By end of the experiment, tumor volumes in control and M-LECP injected groups were nearly identical. In contrast, lymphatic metastasis increased by ~16-fold in M-LECP injected mice, from mean levels of 408x10^4^ (controls) to 6,520x10^4^ (M-LECP injected) RLU/mg of protein (P = 0.02; Fig 11B). The burden of lung metastases also increased 2-fold in M-LECP injected mice although this difference between groups did not reach statistical significance (Fig 11C). This might be because pulmonary lesions are formed after establishment of lymph node metastases so the tumor burden in the lungs might not have achieved maximal level at the time of tissue harvest. Collectively, these data show that TLR4-induced experimental M-LECP are capable of generating functional lymphatic vessels *in vivo* as evidenced by increased dissemination of tumor cells to lymph nodes. These data also suggest that naturally generated M-LECP by TLR4-mediated inflammatory pathway play a significant role in tumor spread.

## Discussion

The main findings of this study are: 1) TLR4 activation causes myeloid-lymphatic transition (MLT) of adult human and mouse myeloid cells; 2) MLT reprogramming is initiated and regulated by NF-κB pathway; 3) MLT is associated with upregulation of multiple inflammatory cytokines and receptors possibly required to promote the functions of M-LECP in autocrine, paracrine and chemotactic manners; 4) this process occurs similarly for human and mouse myeloid cells and is independent of T and B lymphocytes; and 5) M-LECP produced *in vitro* by culturing with TLR4 ligands are capable of increasing the density and the function of new lymphatics in mouse models *in vivo*.

### Activation of TLR4/NF-κB pathway induces myeloid cell differentiation into M-LECP

We report, for the first time, that TLR4 is a significant regulator of lymphatic endothelial cell lineage development in adults through differentiation of myeloid cells into M-LECP. The main supportive evidence is based on substantial increase in expression of multiple LEC-specific genes in human and mouse myeloid cells treated with either LPS, a native TLR4 ligand [39], or two structurally unrelated LPS mimetics (Table 1 and S3 Table). Expression of inflammatory and LEC-specific genes were consistently induced for all three TLR4 ligands (LPS, HMGB1 and nab-paclitaxel) although some differences in profiles of downstream targets were noted (Figs 4–7). Transcriptional changes that were common to all three TLR4 stimuli were likely responsible for M-LECP function determined in mouse models *in vivo* (Figs 9–11).

The pro-lymphatic role of TLR4 is consistent with previous reports of LPS- and HMGB1-induced inflammatory lymphangiogenesis [22,34,40], paclitaxel-induced tumor lymphangiogenesis [38], and decreased ability of TLR4-deficient mice to form lymphatic vessels compared with wild-type [35]. Both LPS- and paclitaxel-induced lymphangiogenesis have been linked to massive recruitment of BM-derived CD11b^+^ myeloid cells expressing LEC-specific markers [22,34,38]. This is consistent with our hypothesis that inflammatory and tumor lymphangiogenesis directly depends on mobilized M-LECP.

Generation of functional LECP *in vitro* was previously reported to require endothelial growth-promoting factors such as VEGF-A [25,27] and/or VEGF-C [24,27]. This implies that myeloid precursors are competent to respond to these factors by expressing functional receptors for VEGF-A and VEGF-C. In our experience, both human CD14^+^ and mouse CD11b^+^ naïve cells express little or no VEGFR-3 prior to TLR4 activation (Figs 1A and 2A). In contrast, the level of VEGFR-3 is significantly increased after stimulation with TLR4 ligands (Figs 1B, 2B, 2C and 2D). This is consistent with our previous finding that mouse RAW264.7 cells completely lack VEGFR-3 but this receptor is highly upregulated after treatment with LPS [22]. The RAW264.7 model also showed that VEGFR-3 and co-expressed VEGF-C create an autocrine loop necessary for acquisition of lymphatic phenotype [22]. Here we show that primary human and mouse myeloid cells also co-express VEGFR-3 and VEGF-C (S3, S4 and S5 Tables). This is consistent with several independent studies that recorded coincident upregulation of VEGFR-3 and VEGF-C during early stages of LECP differentiation [13,24,25,54]. It is well-established that VEGFR-3 and VEGF-C are downstream targets of NF-κB [48,55], which is the main transcriptional mediator of the TLR4 signaling [55,56]. Taken together, these data suggest that TLR4 is one of the earliest inflammatory events that triggers myeloid-lymphatic reprogramming by upregulating VEGFR-3/VEGF-C pair. This conclusion is directly supported by the present study demonstrating effective blockade of the entire myeloid-lymphatic reprogramming by three independent inhibitors of the NF-κB pathway (Fig 8).

### TLR4-induced inflammatory cytokines may promote M-LECP differentiation and functions

While the three TLR4 ligands used here generated distinct gene profiles (S3 Table), all three consistently upregulated some inflammatory cytokines and corresponding receptors. This overlap suggests these specific cytokines and receptors might be instrumental for promoting MLT, supporting M-LECP recruitment to the inflamed tissues or functions of recruited progenitors. For instance, all TLR4 ligands caused co-expression of CCL3 and its receptor, CCR1 (Fig 5) implying a possible autocrine function of this pair in LEC differentiation. In contrast, CXCL1, IL-3 and Ang1 were strongly induced in the absence of corresponding receptors (Fig 5A, 5D and 5G) suggesting these soluble factors are needed for paracrine communication with the environment. Yet, in another group, only receptors were significantly increased (e.g., PDGFRA and CCR8) suggesting that corresponding ligands, PDGF and CCL1, often overexpressed in cancers [57,58], may create a chemokine gradient facilitating recruitment of M-LECP. In summary, our analyses identified a panel of plausible inflammatory regulators of M-LECP that could serve as biomarkers or therapeutic targets for controlling this process.

### Human and mouse myeloid cells can be differentiated into M-LECP using TLR4 ligands

Although LECP were reported to derive from adipose [31], mesenchymal [30] or hematopoietic [59] stem cells, the majority of studies identified BM-produced myeloid-monocytic precursors as their main source [26]. Consistent with the monocytic origin, human LECP were successfully differentiated *in vitro* using monocytes from peripheral [27,32,33] or umbilical cord [24,54] blood and treatment with a VEGF-A/VEGF-C cocktail. Here, we provide evidence that mouse and human myeloid-monocytic cells can undergo similar pro-lymphatic reprogramming upon activation of TLR4. Similarities between human CD14^+^ and mouse CD11b^+^ cell differentiation are indicated by: 1) upregulation of core lymphatic genes (e.g., LYVE1, PDPN, VEGFR3) by all tested TLR4 ligands; 2) increased expression of several transcriptional regulators in both species; 3) similar overlap in upregulation of inflammatory genes; and 4) lack of appreciable effects on PECAM1 and TIE2 that are typically expressed at higher levels on BEC compared with LEC.

We also noticed that several hallmarks of MLT were conserved independently of species, mouse strains or immune status. For instance, PDPN and VEGF-C are typically among the highest upregulated genes, LYVE-1 is upregulated modestly, and VEGFR-1 or VEGF-D are relatively unchanged. This gene expression pattern was similar for myeloid cells from C57BL/6 mice and immunodeficient SCID mice on the BALB/c background (Fig 6) suggesting that MLT is not restricted to a particular mouse strain, and is independent of T and B lymphocytes. These conclusions are important for both understanding the basic tenets of LECP differentiation and practical reasons. Similar patterns for human and mouse MLT established by our studies support extrapolation of conclusions from mouse models to human pathologies. Close reproduction of MLT in immunodeficient mice shown here provides rational basis for *in vivo* modeling of M-LECP generation in the presence of human cancers or other tissues xenografted into SCID mice. Parallel behavior of myeloid cells from different mouse strains and human monocytes strengthens the idea of a predominant myeloid source for LECP. Collectively, these new findings significantly expand experimental approaches for analyzing MLT *in vivo* and determining significance of M-LECP for pathogenesis of human diseases.

### Experimentally-produced and native M-LECP have similar LEC marker signature and lymphangiogenic potential *in vivo*

One of the main goals of this study was to determine whether TLR4-induced *in vitro* differentiated LECP from primary myeloid cells have similar structural and biological properties to naturally-produced progenitors. To date, only a few studies have established the functional competence of *in vitro* differentiated LECP as indicated by the ability to increase functional lymphatic vessels *in vivo*. Here, we generated lymphatic progenitors in culture and tested their functional impact on inflamed lymphatic vasculature in mice. We found that experimentally-produced M-LECP express lymphatic-identifying markers (Fig 10A), and reproduce the biological behavior of native M-LECP (Fig 9). Prior studies determined that endogenous M-LECP are characterized by the following traits: 1) chemo-attraction to tumor [29,53] and inflamed sites [14,22]; 2) co-expression of LEC-specific markers such as Vegfr-3, Lyve-1 or Pdpn and myeloid markers such as CD11b, CD14 or CD68 [16,60]; 3) preferential integration into lymphatic [12,13,22] as opposed to blood vessels [12]; and 4) the ability to increase the density [13,24,32] and the function of lymphatic vessels [25]. Fig 10 shows that *in vitro* TLR4-differentiated M-LECP faithfully display all these traits. Moreover, Fig 11 shows significant increase in lymph node metastasis in mice injected with experimental M-LECP attesting to their ability to augment lymphatic function. This evidence demonstrates that TLR4 activation of myeloid cells produces functional lymphatic progenitors and highlights the role of these progenitors in pathological conditions such cancer.

In summary, we describe a novel protocol for generation of functionally competent lymphatic endothelial progenitors from primary human and mouse myeloid cells. Myeloid-lymphatic transition (MLT) is accompanied by substantial elevation of inflammatory mediators that possibly required for reprogramming, trafficking and/or the functions of M-LECP. MLT is not dependent on lymphoid cells and occurs similarly in myeloid cells from different mouse strains and human monocytes. We believe this study will serve as the foundation for future analyses of M-LECP differentiation and functions leading to better understanding of adult lymphangiogenesis and novel therapeutic approaches for correcting aberrant lymphatic function in human patients.

## Supporting information

S1 Fig Differentiated M-LECP acquire the capacity to perform endothelial-specific functions. Mouse CD11b^+^ isolated from BM were analyzed at three timepoints: *ex vivo*, after 6 days of treatment with CSF1 alone, and after four days with 50 ng/ml LPS for (A) the ability to uptake acetylated-LDL, (B) bind UEA-1 lectin, and (C) bind Tomato lectin. Representative histograms from each time point are presented. The MFI (x10^3^) are present in bar graphs displaying the mean ±SEM.
(PDF)

S2 Fig LPS-differentiated mouse M-LECP perform uptake of acetylated LDL and express endothelial-specific receptors for UEA-1 and Tomato lectins. Mouse CD11b^+^ cells were pretreated with CSF1 followed by 4 days exposure to LPS (50 ng/ml). Differentiated cells were incubated for 4 hours with FITC-tagged (A) 10μg/ml of AC-LDL, (B) 10μg/ml of UEA1 lectin or (C) 10μg/ml of Tomato lectin. Bright fields and fluorescent images are shown side-by-side to show that nearly all cells are able to uptake the compounds. All images were acquired at 200X magnification.
(PDF)

S3 Fig Human CD14^+^ monocytes are positive for proliferative markers. Human CD14^+^ monocytes were isolated from whole blood and plated on slides. Slides were stained daily for evidence of proliferation with (A) acetylated histone H3 (Ac-histone) and (B) Ki-67 for 6 days. White arrowheads point to double nuclei. All images were acquired at 400X magnification.
(PDF)

S1 Table Sequences of human primers used for RT-qPCR. (PDF)

S2 Table Sequences of mouse primers used for RT-qPCR. (PDF)

S3 Table Changes in expression in TLR4-activated human CD14^+^ monocytes undergoing lymphatic reprogramming. (PDF)

S4 Table TLR4-activated lymphatic reprogramming of bone marrow myeloid cells derived from C57BL/6 mice. (PDF)

S5 Table TLR4-activated lymphatic reprogramming 0f bone marrow myeloid cells derived from CB-17/SCID mice. (PDF)
