# Supplementary figures and images for: Lymphatic endothelial progenitors originate from plastic myeloid cells activated by toll-like receptor-4
Source: PLoS One. 2017 Jun 9;12(6):e0179257. doi: 10.1371/journal.pone.0179257 (PMC5466303; doi:10.1371/journal.pone.0179257)

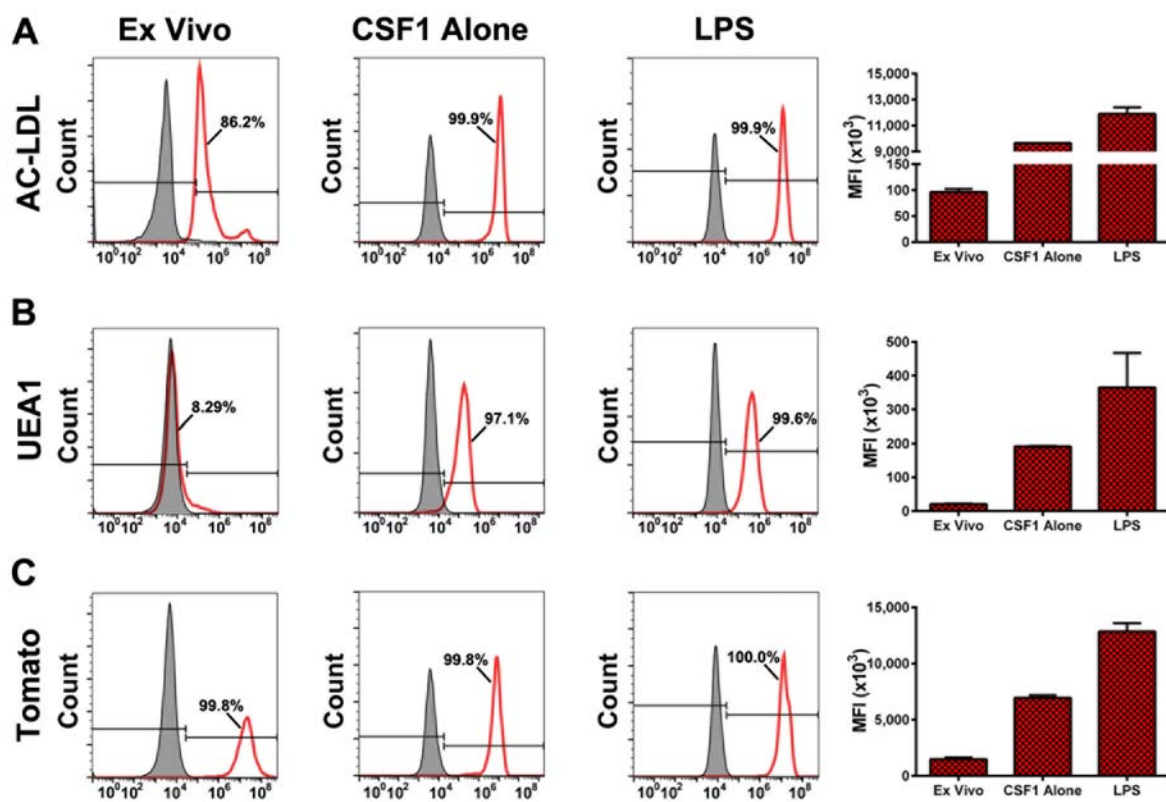

Supplement: S1 Fig — Mouse CD11b+ isolated from BM were analyzed at three timepoints: ex vivo, after 6 days of treatment with CSF1 alone, and after four days with 50 ng/ml LPS for (A) the ability to uptake acetylated-LDL, (B) bind UEA-1 lectin, and (C) bind Tomato lectin. Representative histograms from each time point are presented. The MFI (x103) are present in bar graphs displaying the mean ±SEM. (PDF) [file pone.0179257.s001.pdf]

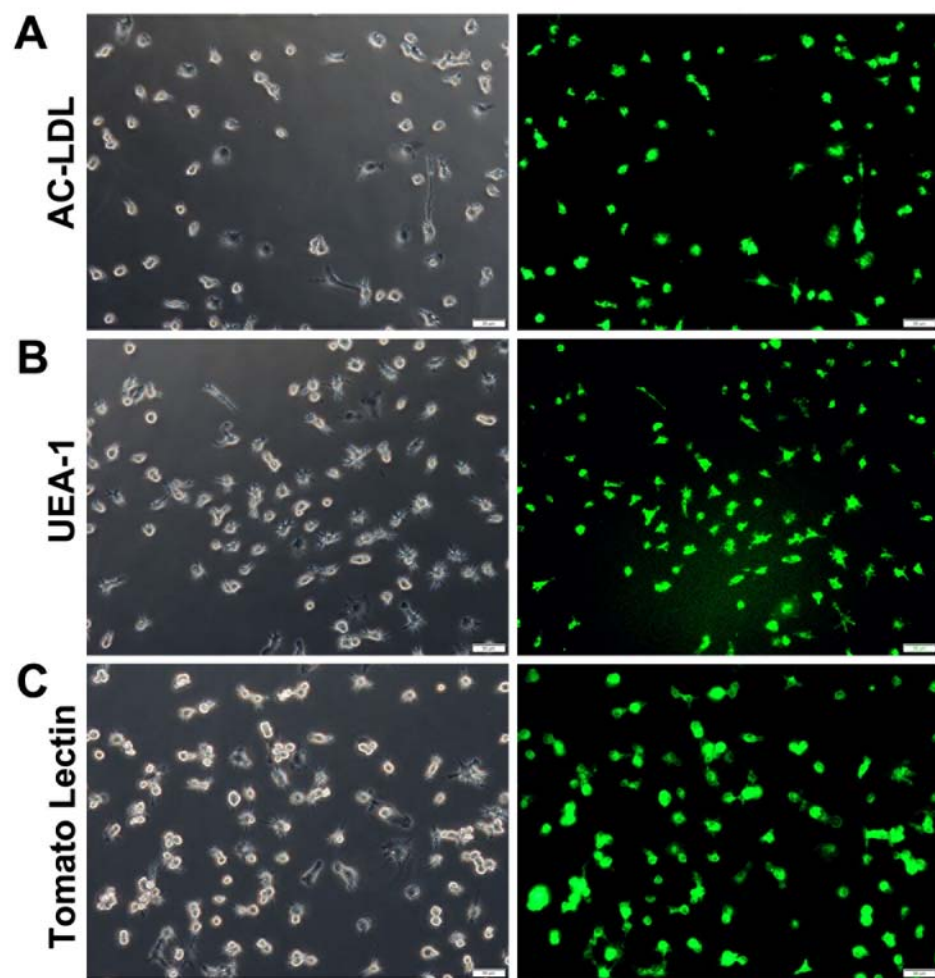

Supplement: S2 Fig — Mouse CD11b+ cells were pretreated with CSF1 followed by 4 days exposure to LPS (50 ng/ml). Differentiated cells were incubated for 4 hours with FITC-tagged (A) 10μg/ml of AC-LDL, (B) 10μg/ml of UEA1 lectin or (C) 10μg/ml of Tomato lectin. Bright fields and fluorescent images are shown side-by-side to show that nearly all cells are able to uptake the compounds. All images were acquired at 200X magnification. (PDF) [file pone.0179257.s002.pdf]

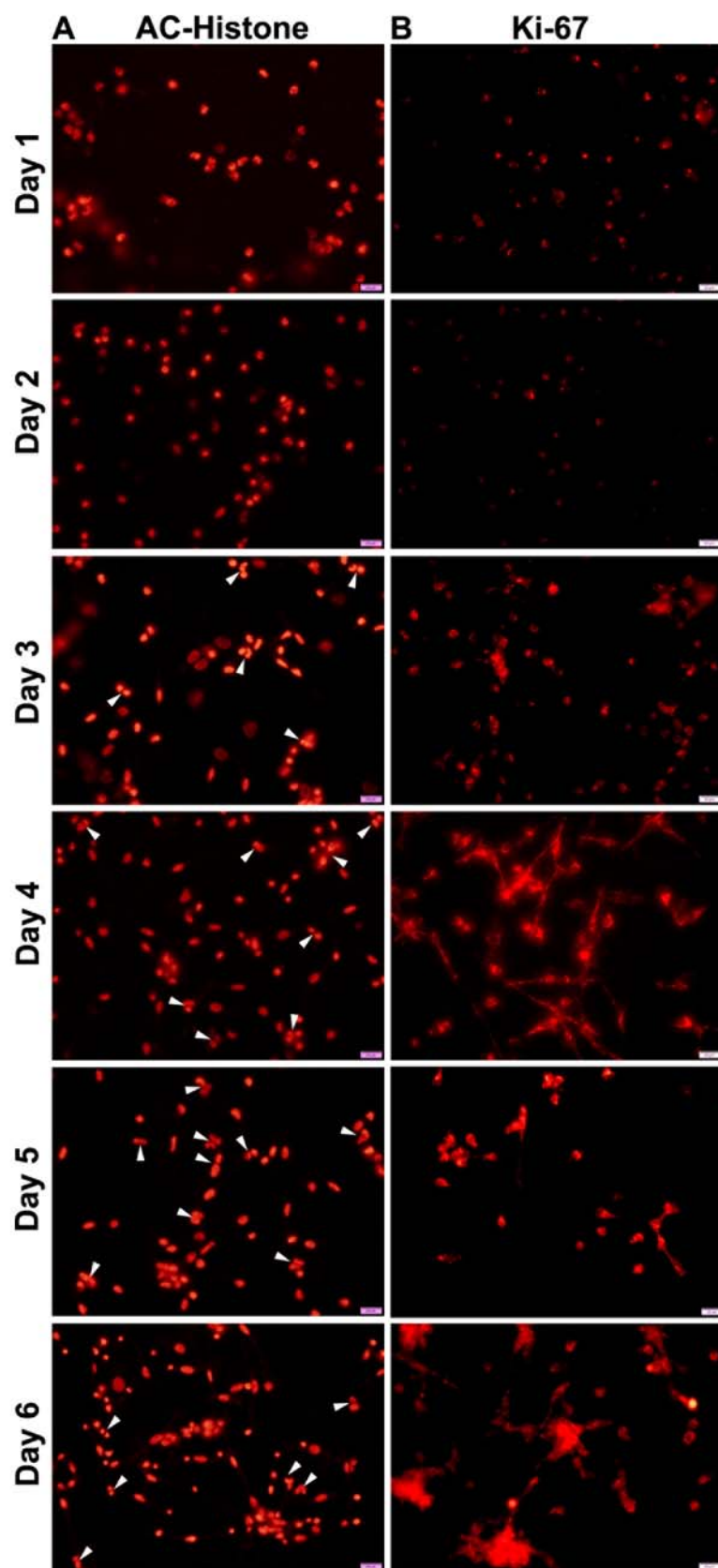

Supplement: S3 Fig — Human CD14+ monocytes were isolated from whole blood and plated on slides. Slides were stained daily for evidence of proliferation with (A) acetylated histone H3 (Ac-histone) and (B) Ki-67 for 6 days. White arrowheads point to double nuclei. All images were acquired at 400X magnification. (PDF) [file pone.0179257.s003.pdf]
