# Supplementary material for: Lymphatic endothelial progenitors originate from plastic myeloid cells activated by toll-like receptor-4
Source: PLoS One. 2017 Jun 9;12(6):e0179257. doi: 10.1371/journal.pone.0179257 (PMC5466303; doi:10.1371/journal.pone.0179257)
Supplement: S1 Table — (PDF) [file pone.0179257.s004.pdf]

S1 Table. Sequences of human primers used for RT-qPCR.

Gene[A]	Product Size (bp)	Forward Primer Sequence (5' → 3')	Reverse Primer Sequence (5' → 3')
ACTB	131	TCCTCTCCCAAGTCCACACAGG	GGGCACGAAGGCTCATCATTC
ANG1	136	CTCGCTGCCATTCTGACTCAC	GACAGTTGCCATCGTGTTCTG
ANG2	73	TCTTGGCCGCAGCCTATAAC	TGCTGGACCTGATATTGCTTCT
BCL2	94	GGGGAGGATTGTGGCCTTC	CAGGGCGATGTTGTCCACC
BCL3	103	ATTGCTGTGGTGCAGGGTAAC	GTGTCTGCCGTAGGTTGTTG
BCL6	76	GGAGTCGAGACATCTTGACTGA	ATGAGGACCGTTTTATGGGCT
BCLXL	119	GAGCTGGTGGTTGACTTTCTC	TCCATCTCCGATTCAGTCCCT
C/EBP	147	ACTCCAGGGGTGAACGGAAT	CATGGGCGAACTCTTTTTGCT
CCL1	141	TCATTTGCGGAGCAAGAGATT	CTGAACCCATCCAACTGTGTC
CCL2	152	CAGCCAGATGCAATCAATGCC	TGGAATCCTGAACCCACTTCT
CCL3	140	AGTTCTCTGCATCACTTGCTG	CGGCTTCGCTTGGTTAGGAA
CCL4	61	CTGTGCTGATCCCAGTGAATC	TCAGTTCAGTTCCAGGTCATACA
CCL5	90	ATCCTCATTGCTACTGCCCTC	GCCACTGGTGTAGAAATACTCC
CCL7	108	AGCTCTCCAGCCTCTGCTTA	CTGCTTTCAGCCCCCAG
CCL17	129	TGGAGCAGTCCTCAGATGTC	CTTCTCTGCAGCACATCCAC
CCL19	114	CCAGCCCCAACTCTGAGTG	ATCCTTGATGAGAAGGTAGTGGA
CCL20	220	TGCTGTACCAAGAGTTTGCTC	CGCACACAGACAACTTTTTCTTT
CCL27	69	CCAGGCTTTCGTGCTTCACC	CTGTGACAGGCTGGGGTTCT
CCL28	129	TGCACGGAGGTTTCACATCAT	ACAGATTCTTCTGCGCTTGAC
CCR1	117	GACTATGACACGACCACAGAGT	TACCAAGGAGTACAGAGGGGG
CCR2	115	AGTTCAGAAGGTATCTCTCGGTG	GGCGTGTTTGTTGAAGTCACT
CCR3	140	GCAATGTGGTGGTGGTGATG	ATGCCCCCTGACATAGTGGAT
CCR4	129	TTTCCAAGCAGACCAAATAC	GCATATACAGCAATTACTAT
CCR5	90	AGGGCTGTGAGGCTTATCTTC	CTGGAAGGTGTTCAGGAGAA
CCR6	139	TTGATTCTGAGATGTTACTG	TTATAAAAAGCAAAGGTGAT
CCR7	98	TCATCTTTGCCATCTACA	TGAGACAGCCTGGACGA
CCR8	114	GTGTGACAACAGTGACCGACT	ACAGGAGGCAATAAAAGACAGC
CCR10	117	ATTACTCTGGGGATGAAGA	CCACGGTCAGGGAGACACT
CDX-2	97	TCCGTGTACACCACTCGATATT	GGAACCTGTGCGAGTGGAT
CD14	122	ACGCCAGAACCTTGTGAGC	GCATGGATCTCCACCTCTACTG
CD24	92	CCTGTTTTTCCTTGCCACAT	GACTCAGGCCAAGAAACGTC
CD33	77	GGCCACTCCAAAAACCTGAC	GACAACCAGGAGAAGATCGGG
CD34	205	TCTACAAATGTATCCTACCA	ACACTGTGCTGATTACAGAGGTCT
CD69	94	ACAGGAACTTGGAAGGACCC	AGAACAGCTCTTTGCATCCG
CD83	96	GAGAAACCTAAGTGGCAAGGTG	AGGACAATCTCCGCTCTGTAT

CD105	107	TGCACTTGGCCTACAATTCCA	AGCTGCCCACTCAAGGATCT
CD133	99	AGTCGGAAACTGGCAGATAGC	GGTAGTGTTGTACTGGGCCAAT
CD146	102	AGCTCCGCGTCTACAAAGC	CTACACAGGTAGCGACCTCC
CEBPG	147	ACTCCAGGGGTGAACGGAAT	CATGGGCGAACTCTTTTTGCT
COUPTF2	129	CGGGTGGTCGCCTTTATGG	ACAGGCATCTGAGGTGAACAG
COX2	194	GGCTTCCATTGACCAGAGCAG	GCCGAGGCTTTTCTACCAGA
CREB1	142	CCACTGTAACGGTGCCAACT	GCTGCATTGGTCATGGTTAATGT
CSF1	228	GCTCTCCCAGGATCTCATCAC	TCAAAGGAACGGAGTTAAAACGG
CSF2	110	AAAGGGGATGACAAGCAGAA	ACTACAAGCAGCACTGCCCT
CSFR1	176	GAATCCCAGTGATAGAGCCCA	TTGGAAGGTAGCGTTGTTGGT
CSFR2	95	TGAAGGAAATTACCTGAGA	TCACAGCATCATCAAGA
CX3CL1	122	ACCACGGTGTGACGAAATG	CTCCAAGATGATTGCGCGTTT
CX3CR1	132	AGTGAGCACTCCCAAGTCTA	AGCATGAATGATGGGGTA
CXCL1	97	CTTCCTCCTCCCTTCTGGTC	CCAAACCGAAGTCATAGCCA
CXCL3	86	CGCCCAAACCGAAGTCATAG	GCTCCCCTTGTTCAGTATCTTTT
CXCL5	259	GGAAAGATTTTGTTGTTGTT	AGTCACCTACAATTCAAGAC
CXCL6	65	AGAGCTGCGTTGCACTTGTT	GCAGTTTACCAATCGTTTTGGGG
CXCL9	93	CCAGTAGTGAGAAAGGGTCGC	TGGGGCAAATTGTTTAAGGTCTT
CXCL10	193	GTGGCATTCAAGGAGTACCTC	GCCTTCGATTCTGGATTCAGACA
CXCL12	101	ATGCCCATGCCGATTCTTCG	GCCGGGCTACAATCTGAAGG
CXCL13	62	TTGAGGTGTAGATGTGTCCAAGA	ATTCGATCAATGAAGCGTCTAGG
CXCR1	127	TGGGGACTGTCTATGAATCTGT	GCAACACCATCCGCCATTTT
CXCR2	249	TGGATTTTTGGCACATTCC	TGGGCTAACATTGGATGAGT
CXCR3	160	GCTGGTGCTGACACTGCCGCT	AACTATGTTCAGGTAGCGGT
CXCR4	63	TACACCGAGGAAATGGGCTCA	TTCTTCACGGAAACAGGGTTC
CXCR5	155	ACCTCCCGATTCCTCTACCAT	AAGATGCTTGTCACCAGGATG
CXCR6	169	GACTATGGGTTCAGCAGTTTCA	GGCTCTGCAACTTATGGTAGAAG
C3a	125	GGGGAGTCCCATGTACTCTATC	GGAAGTCGTGGACAGTAACAG
C5a	104	CAGGGAAGGTTACCGAGCAAT	TCAGATGTTCTCCCACTAGCA
C3aR1	100	CCCTACGGCAGGTTCCTATG	GACAGCGATCCAGGCTAATGG
C5aR1	96	TCCTTCAATTATACCACCCCTGA	ACGCAGCGTGTTAGAAGTTTTAT
DLL4	131	TCCAACTGCCCTTCAATTTCAC	CTGGATGGCGATCTTGCTGA
E2F1	146	ACGTGACGTGTCAGGACCT	GATCGGGCCTTGTTTGCTCTT
ETS1	96	GATAGTTGTGATCGCCTCACC	GTCCTCTGAGTCGAAGCTGTC
FOXC2	134	CCTCCTGGTATCTCAACCACA	GAGGGTCGAGTTCTCAATCCC
FOXN2	124	AGAGAGCTGAAACCCCAGGAG	GCTGACTCACTGTCCACTAGAG
FZD1	261	AGACCGAGTGGTGTGTAATGA	AGGATGGTGATGGTCTTGAT
FZD4	86	TACCTCACAAAACCCCCATCC	GCTCCTTTTCACCCAGATGT

HEMGN	219	AGCTGAAGTGCATGAAAAGGAA	ACTTTGGTTATGCTCCCAGGT
HIF1A	126	ATCCATGTGACCATGAGGAAATG	CTCGGCTAGTTAGGGTACACTT
HIF2A	196	TTGATGTGGAAACGGATGAA	GGAACCTGCTCTTGCTGTTC
HOXA4	141	ATAACGGAGGGGAGCCTAAG	GCTCAGACAAACAGAGCGTG
HOXA9	153	TACGTGGACTCGTTCCTGCT	CGTCGCCTTGGACTGGAAG
IFI16	97	CCTCCATCAACACCAAGCA	TCACTCTGGGCACTGTCTT
IFNG	100	TCGGTAACTGACTTGAATGTCCA	TCCTTTTTCGCTTCCCTGTTTT
IFNGR	108	AAAGTCAGAAGAATTTGCTGTAT	ACTGAAGGGTGAAATATGTC
IL-1B	100	CAGCTACGAATCTCCGACCAC	GGCAGGGAACCAGCATCTTC
IL-3	125	GCGATCTTTTGAGTCCAACG	GCTTCTGGTCCTGGAATGTG
IL-4	150	CCAACTGCTTCCCCCTCTG	TCTGTTACGGTCAACTCGGTG
IL-5	102	CTGCCTACGTGTATGCCATCC	CATTGGCTATCAGCAGAGTTCG
IL-6	159	AACCTGAACCTTCCAAAGATGG	TCTGGCTTGTTCCTCACTACT
IL-7	109	TTGGACTTCCTCCCCTGATCC	TCGATGCTGACCATTAGAACAC
IL-8	112	ACTGAGAGTGATTGAGAGTGGAC	AACCCTCTGCACCCAGTTTTC
IL-10	103	CTCATGGCTTTGTAGATGCCT	GCTGTCATCGATTTCTTCCC
IL-15	129	TTTCAGTGCAGGGCTTCCTAA	GGGTGAACATCACTTTCCGTAT
IL-17A	156	CAATCCCACGAAATCCAGGATG	GGTGGAGATTCCAAGGTGAGG
IL17B	111	ATGAAAGCCTCTAGTCTTGCCT	TGTGGCGATCACACAGCTTC
IL-17D	290	GCCCTGGGCCTACAGAATC	CGCCCTGTTTGTCGATGCT
IL17E	205	ATGTACCAGGTGGTTGCATTC	TGCTGTTGAGGGGTCCATCT
IL-18	75	TCTTCATTGACCAAGGAAATCGG	TCCGGGGTGCATTATCTCTAC
IL-23	111	GGACAACAGTCAGTTCTGCTT	CACAGGGCTATCAGGGAGC
IL-33	190	CAGCAAGCAAAGCCTTGTGTT	GCGAGTACCAGATGTCTTTTGT
IL1R	118	TCCAGTCACTAATTTCCAGAAGC	CCTGTACCAAAGCACAATGTCAA
IL3R	106	CCCTGTCTCCTGCAAACGAAG	CGGTCACATTTCTGTTAAGGTCC
IL4R	110	ATTGTCTACTCAGCCCTTAC	CAGCAGCCACAGCAAGGACT
IL5R	271	GAAGGAACTCGTCTCTCTATCCA	TCAAGGGCTTGTGTTCATCATTT
IL6R	215	GCTCCTCTGCATTGCCATTG	CATCTGGTCGGTTGTGGCT
IL7R	245	GGACGCATGTGAATTTATCCAGC	CAAGATGACCAACAGAGCGAC
IL10R	109	CTTGGCTCAGACGCTCATGG	CAGACTGATTTGGGATGGGTG
IL15R	114	CGCAGACATCTGGGTCAAGAG	TTGTTCAACACGCACTCCGT
IL17RA	159	GTTTTACCTTCAGCCACTTT	ATGGCGTGGTTACCTTCAT
IL17RB	63	CAGCGACTGCATCCGACATAA	GGGAAAGGGACGCCTGTTT
IL17RD	115	AGGCATCTATGACTCGTCTGT	TGAAGAGGAGGACACGCT
IL18R	197	AAGAACGCCGAGTTTGAAGAT	GAGCAGTTGAGCCTTACGTTT
IL23R	115	TGGGTCCAAGCAGCAAA	AATGACGGCTGCAGAAGG
IL33R	182	ATTCCACAGCAGCAAAGTTT	CTGAGTGGGAATACTTTTGT

INOS	116	ATTCTGCTGCTTGCTGAGGT	ATGCAATGAATGGGGAAAAA
IRF3	96	GTGGCCTGGGTGAACAAGAG	CTGGAAGATTCCGAAATCCTCC
IRF5	169	AGGAGAGGAGGAGGAAGAA	ATCTTGTAGGGCTGAGGTG
IRF7	86	TACCATCTACCTGGGCTTC	AGCCAGGGTTCCAGCTTCA
IRF8	77	ATGTGTGACCGGAATGGTGG	AGTCCTGGATACATGCTACTGTC
ITGA9	89	GACGCTGATCCCTTGCTATGA	CGGTGAAGAAGCCCGCTATC
KLF2	137	CTACACCAAGAGTTCGCATCTG	CCGTGTGCTTTCGGTAGTG
KIT	135	GTTCTGCTCCTACTGCTTCGC	TAACAGCCTAATCTCGTCGCC
LTB	103	GTACGGGCCTCTCTGGTACA	GTCCACCATATCGGGGTGAC
LYVE-1	150	TGGGGATCACCCTTGTGAG	AGCCATAGCTGCAAGTTTCAAA
MAF	91	CGAGTGGGCTCAGTTATGAA	ACGAGAAGTTGGTGAGCAGC
MAFB	88	GACGCAGCTCATTCAGCAG	CTCGCACTTGACCTTGTAGGC
MNDA	110	GTTTACTCCGAATCAGGAAACCC	TAAATGGCGCTGTTGCTTTCA
MTOR	114	GCAGATTTGCCAACTATCTTCGG	CAGCGGTAAAAGTGTCCCCTG
NFATc1	119	CACCGCATCACAGGGAAGAC	GCACAGTCAATGACGGCTC
NFKB1	122	TGCCAACAGATGGCCCATAC	TGTTCTTTTCACTAGAGGCACCA
NFKB2	154	TCCGGGGGCATCAAA	AAAGGCCTGCCATCCA
NOTCH1	140	GAGGCGTGGCAGACTATGC	CTTGTACTCCGTCAGCGTGA
NRP1	94	ATCACCCAAGTGAAAAATGCGA	TCCTCCAAATCGAAGTGAGGG
NRP2	65	CCAGCAGCCAAAGCTGTTC	GGTCAAACCTTCGGATGTCAG
PAX4	132	ATACCCGGCAGCAGATTGTG	AAGACACCTGTGCGGTAGTAA
PAX5	127	ACTTGCTCATCAAGGTGTCAG	TCCTCCAATTACCCCAGGCTT
PAX6	111	TGGGCAGGTATTACGAGACTG	ACTCCCGCTTATACTGGGCTA
PDGFB	175	TCTCTGCTGCTACCTGCGT	GTGGGAGCGGGTCATGTTC
PDGFC	128	ACTCAGGCGGAATCCAACC	CTTGGGCTGTGAATACTTCCATT
PDGFRA	126	AACCGTGTATAAGTCAGGGGA	ATTTCTTCCAGCATTGTGAT
PDGFRB	155	TCCAGCACCTTCGTTCTGAC	TATTCTCCCGTGTCTAGCCCA
PECAM1	131	ACAGTGTTGACATGAAGA	ATCCTTATAGAACAGCATC
Podoplanin	128	AGAGCAACAACTCAACGGGA	TGTAGTCTCAGTGTCATCTTC
PROX1	130	GGATGTTGAGTATTCAGTGGTGC	CTGGGAAATTATGGTTGCTCCT
RELA	96	CCCACGAGCTTGTAGGAAAGG	GGATTCCCAGGTTCTGGAAAC
RELB	91	CAGCCTCGTGGGGAAAGAC	GCCCAGGTTGTTAAAACTGTGC
RELC	97	GCAGAGGGGAATGCGTTTTAG	AGAAGGGTATGTTCGGTTGTTG
RUNX2	101	TGGTTACTGTCATGGCGGGTA	TCTCAGATCGTTGAACCTTGCTA
SAA	89	GCTTCTTTTCGTTCCTTGGCG	GCCGATGTAATTGGCTTCTCTCA
SIX1	135	CTGCCGTCGTTTGGCTTTAC	GCTCTCGTTCTTGTGCAGGT
SLP76	100	GTTTGCGTGTGAAGATGCTC	GACATCCAGAAGTTCCCCAA
SOX7	110	CTCTTCTGGGACAGCGTCA	GCCAAGGACGAGAGGAAAC

SOX17	94	GTGGACCGCACGGAATTTG	GGAGATTCACACCGGAGTCA
SOX18	109	GTTCAGCTCCTTCCACGCTT	CTTCATGGTGTGGGCAAAG
SPI1	97	GTGCCCTATGACACGGATCTA	AGTCCCAGTAATGGTCGCTAT
SPRED1	83	CAGCCAGGCTTGGACATTCA	TGGGACTTTAGGCTTCCACAT
SPRED2	114	TATATTGTGCGTGTCAAGGCTG	GGGGTGCATGACCTTACAGA
SP140	123	AGGATGGTCGCAGAGATCCA	TGGCCTTGTTATTGCACTTGC
SYK	110	TTCCCATCCTGCGTCCT	TCTGGGGGCCTTTGTCT
T, Brachyury	109	TATGAGCCTCGAATCCACATAGT	CCTCGTTCTGATAAGCAGTCAC
TAL1	104	AGCCGGATGCCTTCCCTAT	CCGCACAACTTTGGTGTGG
TCF7	92	CTGGCTTCTACTCCCTGACCT	ACCAGAACCTAGCATCAAGGA
TEAD2	138	GCCTCCGAGAGCTATATGATCG	TCACTCCGTAGAAGCCACCA
TIE2	105	TTAGCCAGCTTAGTTCTCTGTGG	AGCATCAGATACAAGAGGTAGGG
TLR1	107	ATTTCAAACGTGAAGCTACAGGG	CCGAACACATCGCTGACAACT
TLR2	163	ATCCTCCAATCAGGCTTCTCT	ACACCTCTGTAGGTCACTGTTG
TLR3	187	TACCAGCCGCCAACTTCAC	AGTTCAGTCAAATTCGTGCAGAA
TLR4	78	TACAAAATCCCCGACAACCTCC	GCTGCCTAAATGCCTCAGGG
TLR5	218	AGCCCCTACAAGGGAAAACG	GTGGTGGGCAAGAATCAAAGA
TLR6	211	TCTGAATGCAAAAACCCTTCACC	CCAAGTCGTTTCTATGTGGTTGA
TLR7	124	CGAACACCACGAACCTCACC	CCCAGTGGAATAGGTACACAGTT
TLR8	142	TGTTCCTTCAGTCGTCAATGC	TTGCTGCACTCTGCAATAACT
TLR10	211	AGTGGAACACTTTCAGATCCGA	GTGTGGCATTTGTGCATTTGATA
TNF	91	GAGGCCAAGCCCTGGTATG	CGGGCCGATTGATCTCAGC
TNFR	166	TCACCGCTTCAGAAAACCACC	AAGCACTGGAAAAGGTTTT
TRIF	169	GGCCCATCACTTCCTAGCG	GAGAGATCCTGGCCTCAGTTT
VEGF-A	117	ATCACGAAGTGGTGAAGTTC	TGCTGTAGGAAGCTCATCTC
VEGF-B	114	TGCAGATCCTCATGATCCG	TGTCTGGCTTCACAGCAC
VEGF-C	91	CCCCAAACCAGTAACAATC	CACAGGCACATTTTCCAG
VEGF-D	128	TCGCTGTTCCCATTCCAAGAAAC	CTGGTTCCTGGAGATGAGAGTGGTC
VEGFR-1	109	AGCAGGTGCTTGAAACCGTAG	GTCGCAGGTAACCCATCTTTT
VEGFR-2	105	GGCCCAATAATCAGAGTGGCA	TGTCATTTCCGATCACTTTTGGA
VEGFR-3	259	GCACTGCCACAAGAAGTACCT	GCTGCACAGATAGCGTCCC
WNT3A	110	ATGGCCCCACTCGGATACTT	GAGCCCAGGGAGGAATACTG
ZNF292	162	AGCTGTGCCAGACACTCCTA	CCAAGCGTTCCAGAACCAAG

[A] Primers were designed on human CDS of targets found in NCBI database and validated using human universal cDNA. Primers were confirmed to exclusively detect species-specific cDNA.
