# Supplementary material for: Lymphatic endothelial progenitors originate from plastic myeloid cells activated by toll-like receptor-4
Source: PLoS One. 2017 Jun 9;12(6):e0179257. doi: 10.1371/journal.pone.0179257 (PMC5466303; doi:10.1371/journal.pone.0179257)
Supplement: S2 Table — (PDF) [file pone.0179257.s005.pdf]

S2 Table. Sequences of mouse primers used for RT-qPCR.

Gene[A]	Product Size (bp)	Forward Primer Sequence (5' → 3')	Reverse Primer Sequence (5' → 3')
Actb	153	GGCTGTATTCCCCTCCATCG	CCAGTTGGTAACAATGCCATGT
Ccl2	108	ATTGGGATCATCTTGCTGGT	CCTGCTGTTCACAGTTGCC
Ccl3	91	GTGGAATCTTCCGGCTGTAG	ACCATGACACTCTGCAACCA
Ccl19	120	TAATGATGCGGAAGACTGCT	GTGAACACAACAGCAGGCAC
Ccl20	180	TTTTCACCCAGTTCTGCTTT	AGGCAGAAGCAAGCAACTAC
Ccr1	142	CTCATGCAGCATAGGAGGCTT	ACATGGCATCACCAAAAATCCA
Ccr3	195	TCAACTTGGCAATTTCTGACCT	CAGCATGGACGATAGCCAGG
Ccr6	179	CTTTGGAACGGATGATTATG	TGTAGAAGGCAAAGGTCAT
CD14	191	CTCTGTCCTTAAAGCGGCTTAC	GTTGCGGAGGTTCAAGATGTT
CD33	139	CCGCTGTTCTTGCTGTGTG	AAGTGAGCTTAATGGAGGGGTA
CD105	155	AGGGGTGAGGTGACGTTTAC	GTGCCATTTTGCTTGGATGC
CD133	81	CTCCCATCAGTGGATAGAGAACT	ATACCCCCTTTTGACGAGGCT
CD146	220	CCCAAACTGGTGTGCGTCTT	GGAAAATCAGTATCTGCCTCTCC
Cdx2	243	TACCCGGACTACGGTGGTTAC	GTGATGGTGCGCGTGGTAT
Csf1r	121	TGTCATCGAGCCTAGTGGC	GGTCCAAGGTCCAGTAGGG
Cxcl1	168	GCTGGGATTCACCTCAAGAA	TGGGGACACCTTTTAGCATC
Cx3cl1	120	ACGAAATGCGAAATCATGTGC	CTGTGTCGTCTCCAGGACAA
C5ar1	80	CATACCTGCGGATGGCATTCA	GGAACACCACCGAGTAGATGAT
E2f1	97	GAGAAGTCACGCTATGAAACCTC	CCCAGTTCAGGTCAACGACAC
Hoxa4	75	CGGTGGTGTACCCCTGGAT	GCTTAGGTTCGCCTCCGTTAT
Il-10	158	GCCTTATCGGAAATGATCCA	TTTTCACAGGGGAGAAATCG
Il-15	112	CATCCATCTCGTGCTACTTGTG	GCCTCTGTTTTAGGGAGACCT
Ifngr	149	CTGGCAGGATGATTCTGCTGG	GCATACGACAGGGTTCAAGTTAT
Irf7	178	GCGTACCCTGGAAGCATTTC	GCACAGCGGAAGTTGGTCT
Itga9	208	AAGTGTCGTGTCCATACCAAC	GGTCTGCTTCGTAGTAGATGTTC
Lyve-1	112	CAGCACACTAGCCTGGTGTTA	CGCCCATGATTCTGCATGTAGA
Maf	104	TGTCTCTGCTGCACCCTCTTG	AGCAAGGAGGAGGTGATCCG
Mafb	179	TTCGACCTTCTCAAGTTCGACG	TCGAGATGGGTCTTCGGTTCA
Notch1	162	CCCTTGCTCTGCCTAACGC	GGAGTCCTGGCATCGTTGG
Pax6	194	TACCAGTGTCTACCAGCCAAT	TGCACGAGTATGAGGAGGTCT
Pecam-1	120	TCACCATCAACAGCATCCAT	GGTGCTGAGACCTGCTTTTC
Podoplanin	159	ACCGTGCCAGTGTTGTTCTG	AGCACCTGTGGTTGTTATTTTGT
Saa	128	CCCGAGCATGGAAGTATTTG	AGTCTGCCATGGAGGGTTTT
Six1	148	ATGCTGCCGTCGTTTGGTT	CCTTGAGCACGCTCTCGTT

Syk	103	CTACCTGCTACGCCAGAGC	GCCATTAAGTTCCCTCTCGATG
Tead2	148	GAAGACGAGAACGCGAAAGC	GATGAGCTGTGCCGAAGACA
Tie2	67	CGGCCAGGTACATAGGAGGAA	TCACATCTCCGAACAATCAGC
Tlr2	231	GCAAACGCTGTTCTGCTCAG	AGGCGTCTCCCTCTATTGTATT
Tlr4	129	ATGGCATGGCTTACACCACC	GAGGCCAATTTTGTCTCCACA
Vegfr-1	163	CTCAGACAAGTCAAACCTGGAG	GGGAACTTCATCTGGGTCCATAA
Vegfr-2	133	TTTGGCAAATACAACCCTTCAGA	GCAGAAGATACTGTCACCACC
Vegfr-3	182	CTGGCAAATGGTTACTCCATGA	ACAACCCGTGTGTCTTCACTG
Vegfc	160	GAGGTCAAGGCTTTTGAAGGC	CTGTCCTGGTATTGAGGGTGG
Vegfd	67	GAGGACTGGAAGCTGTGGCG	GCGTGAGTCCATACTGGCAAGA

[A] Primers were designed based on mouse CDS of targets found in NCBI database and validated using mouse universal cDNA. Primers were confirmed to exclusively detect species-specific cDNA.
