# Supplementary material for: Lymphatic endothelial progenitors originate from plastic myeloid cells activated by toll-like receptor-4
Source: PLoS One. 2017 Jun 9;12(6):e0179257. doi: 10.1371/journal.pone.0179257 (PMC5466303; doi:10.1371/journal.pone.0179257)
Supplement: S3 Table — (PDF) [file pone.0179257.s006.pdf]

S3 Table. Changes in expression in TLR4-activated human CD14$^+$ monocytes undergoing lymphatic reprogramming[A].

**Category**	**Gene[B]**	**LPS[C]**	**HMGB1[C]**	**Nab-PXL[C]**
**Cell progenitor**	**CD24**	1.98 ± 0.04[D]	2.76 ± 0.05	1.71 ± 0.03
	**CD33**	25.06 ± 0.66	20.54 ± 0.41	15.29 ± 0.57
	**CD34**	6.77 ± 0.32	5.82 ± 0.26	4.03 ± 0.08
	**CD133**	12.16 ± 0.36	7.08 ± 0.19	4.59 ± 0.14
	**CD146**	167.82 ± 5.21	134.43 ± 4.08	102.32 ± 1.98
	**DLL4**	7.71 ± 0.18	3.96 ± 0.10	1.97 ± 0.03
	**KIT**	14.90 ± 0.42	7.24 ± 0.10	2.66 ± 0.04
	**NOTCH1**	73.21 ± 2.15	42.52 ± 0.59	20.36 ± 0.84
**Lymphatic and Endothelial**	**CD105**	867.61 ± 29.52	662.01 ± 20.17	199.60 ± 7.16
	**ITGA9**	407.69 ± 16.84	301.86 ± 11.18	94.45 ± 4.15
	**LYVE-1**	28.78 ± 0.75	26.49 ± 0.81	15.29 ± 0.55
	**NRP1**	76.49 ± 2.00	32.38 ± 0.85	9.70 ± 0.28
	**NRP2**	5.17 ± 0.10	1.89 ± 0.05	2.66 ± 0.05
	**PECAM1**	2.88 ± 0.11	4.64 ± 0.16	2.09 ± 0.14
	**Podoplanin**	899.78 ± 10.27	508.72 ± 15.66	225.02 ± 6.05
	**PROX1**	3.11 ± 0.14	2.66 ± 0.08	2.44 ± 0.06
	**SLP76**	1.21 ± 0.05	1.17 ± 0.01	0.95 ± 0.01
	**SPRED1**	1.89 ± 0.05	1.61 ± 0.13	1.22 ± 0.03
	**SPRED2**	3.79 ± 0.11	2.25 ± 0.04	1.14 ± 0.04
	**VEGFR-1**	7.45 ± 0.14	2.78 ± 0.08	1.93 ± 0.08
	**VEGFR-2**	16.08 ± 0.31	5.17 ± 0.07	2.53 ± 0.10
	**VEGFR-3**	142.70 ± 2.07	115.68 ± 3.54	73.21 ± 2.01
	**VEGF-A**	81.80 ± 2.36	73.53 ± 1.54	46.71 ± 2.52
	**VEGF-B**	13.72 ± 1.07	9.58 ± 0.13	4.41 ± 0.16
	**VEGF-C**	410.49 ± 16.44	194.09 ± 5.34	129.50 ± 1.42
	**VEGF-D**	13.28 ± 0.42	2.96 ± 0.12	2.46 ± 0.06
**Transcription Factors**	**BCL3**	5.33 ± 0.04	3.96 ± 0.12	1.63 ± 0.05
	**C/EBP**	0.89 ± 0.03	0.86 ± 0.03	0.66 ± 0.05
	**CCEB1**	4.08 ± 0.08	2.29 ± 0.04	2.18 ± 0.03
	**CDX-2**	18.21 ± 0.21	17.28 ± 0.71	13.94 ± 0.55
	**COUPTFII**	6.53 ± 0.16	3.34 ± 0.02	5.29 ± 0.12

	**E2F1**	206.07 ± 4.11	161.66 ± 2.34	78.87 ± 3.34
	**ETS1**	2.51 ± 0.04	1.41 ± 0.02	1.71 ± 0.06
	**FOXC2**	7.93 ± 0.25	7.06 ± 0.14	4.74 ± 0.13
	**HIF1A**	3.33 ± 0.04	2.52 ± 0.09	1.37 ± 0.03
	**HIF2A**	6.25 ± 0.14	4.61 ± 0.08	1.56 ± 0.02
	**HOXA4**	236.22 ± 6.81	92.44 ± 2.10	84.45 ± 0.93
	**HOXA9**	10.32 ± 0.12	32.54 ± 1.06	5.45 ± 0.25
	**IRF3**	2.98 ± 0.07	1.66 ± 0.07	4.48 ± 0.12
	**IRF5**	2.96 ± 0.05	2.20 ± 0.07	9.52 ± 0.41
	**IRF7**	29.05 ± 0.74	23.65 ± 0.72	67.36 ± 1.67
	**IRF8**	6.62 ± 0.60	3.00 ± 0.07	14.16 ± 1.35
	**KLF2**	1.56 ± 0.07	1.31 ± 0.03	1.20 ± 0.05
	**MAF**	9.70 ± 0.40	8.78 ± 0.20	4.32 ± 0.10
	**MAFB**	30.72 ± 1.05	35.43 ± 0.81	32.77 ± 1.05
	**NFATc1**	8.13 ± 0.25	3.60 ± 0.08	2.43 ± 0.07
	**NFKB1**	10.33 ± 0.59	7.65 ± 0.32	5.05 ± 0.07
	**NFKB2**	8.76 ± 1.52	5.29 ± 0.10	3.41 ± 0.05
	**PAX4**	2.08 ± 0.06	1.54 ± 0.04	2.07 ± 0.05
	**PAX6**	121.14 ± 3.11	98.64 ± 3.01	70.23 ± 2.13
	**RELA**	10.23 ± 0.45	3.62 ± 0.14	2.67 ± 0.05
	**SIX1**	224.09 ± 9.19	109.39 ± 0.69	172.10 ± 4.16
	**SOX7**	1.85 ± 0.06	1.83 ± 0.07	1.48 ± 0.01
	**SOX17**	2.10 ± 0.04	1.80 ± 0.07	1.29 ± 0.02
	**SOX18**	20.12 ± 0.44	17.35 ± 0.48	10.25 ± 0.36
	**TAL1**	2.51 ± 0.04	1.37 ± 0.03	1.70 ± 0.07
	**TEAD2**	217.83 ± 5.02	80.69 ± 2.80	186.16 ± 4.43
**Cytokines**	**ANG1**	34.63 ± 0.97	12.86 ± 0.46	4.32 ± 0.18
	**ANG2**	15.18 ± 0.40	11.42 ± 0.26	5.22 ± 0.06
	**C3a**	36.39 ± 1.94	4.03 ± 0.17	21.17 ± 0.65
	**C5a**	120.58 ± 8.65	28.41 ± 2.35	6.24 ± 0.73
	**CCL1**	0.89 ± 0.03	0.86 ± 0.03	0.66 ± 0.05
	**CCL2**	66.49 ± 3.23	38.34 ± 1.33	34.88 ± 1.25
	**CCL3**	121.43 ± 3.67	33.06 ± 0.84	48.86 ± 1.48
	**CCL4**	30.42 ± 0.74	16.88 ± 1.22	4.68 ± 0.15
	**CCL5**	8.44 ± 0.07	12.36 ± 0.35	6.90 ± 0.14
	**CCL7**	2.47 ± 0.05	2.13 ± 0.06	1.26 ± 0.04
	**CCL17**	15.27 ± 0.84	7.09 ± 0.80	3.56 ± 0.31

	**CCL19**	206.07 ± 4.11	161.66 ± 2.34	78.87 ± 3.34
	**CCL20**	105.20 ± 2.10	21.47 ± 0.77	47.20 ± 1.58
	**CCL27**	42.57 ± 3.48	26.21 ± 1.41	9.78 ± 0.64
	**CCL28**	13.36 ± 0.89	5.76 ± 0.39	2.28 ± 0.08
	**CSF1**	385.53 ± 11.70	39.22 ± 0.69	6.40 ± 0.14
	**CSF2**	33.77 ± 4.68	11.92 ± 0.56	2.85 ± 0.10
	**CXCL1**	431.62 ± 8.35	119.48 ± 3.29	299.59 ± 4.38
	**CXCL3**	275.02 ± 2.31	51.75 ± 0.87	16.57 ± 0.23
	**CXCL5**	1.55 ± 0.05	0.51 ± 0.02	0.34 ± 0.01
	**CXCL6**	16.30 ± 0.23	16.49 ± 0.45	4.63 ± 0.19
	**CXCL8**	34.99 ± 2.96	10.02 ± 0.84	3.17 ± 0.16
	**CXCL9**	6.76 ± 0.16	0.93 ± 0.05	4.17 ± 0.09
	**CXCL10**	7.06 ± 0.06	5.67 ± 0.19	1.93 ± 0.09
	**CXCL12**	300.98 ± 4.97	50.14 ± 2.07	15.40 ± 0.75
	**CXCL13**	43.14 ± 3.19	18.68 ± 0.37	10.41 ± 0.11
	**CX3CL1**	0.53 ± 0.02	1.24 ± 0.46	2.00 ± 0.08
	**IL-1B**	136.73 ± 6.78	10.36 ± 0.18	26.06 ± 0.38
	**IL-3**	32.10 ± 1.15	20.70 ± 0.87	28.12 ± 0.68
	**IL-4**	20.21 ± 0.28	8.72 ± 0.41	4.23 ± 0.19
	**IL-5**	244.24 ± 13.17	73.20 ± 1.62	19.79 ± 0.11
	**IL-6**	131.95 ± 3.40	75.78 ± 8.96	20.49 ± 0.51
	**IL-7**	6.90 ± 0.08	2.58 ± 0.09	2.01 ± 0.35
	**IL-8**	69.91 ± 2.23	54.84 ± 1.25	32.23 ± 0.74
	**IL-10**	106.89 ± 0.59	8.76 ± 0.22	52.27 ± 2.09
	**IL-15**	244.65 ± 10.04	15.86 ± 0.31	55.09 ± 1.33
	**IL-17A**	1.25 ± 0.02	1.14 ± 0.02	1.36 ± 0.05
	**IL-17B**	40.78 ± 2.65	9.97 ± 0.80	2.07 ± 0.12
	**IL-17D**	3.70 ± 0.11	3.67 ± 0.13	2.90 ± 0.05
	**IL-17E**	6.84 ± 0.66	4.07 ± 0.18	1.31 ± 0.13
	**IL-18**	15.86 ± 0.35	10.98 ± 0.24	6.75 ± 0.34
	**IL-19**	50.23 ± 1.32	34.23 ± 0.76	14.10 ± 0.40
	**IL-23a**	14.98 ± 0.70	3.22 ± 0.15	2.23 ± 0.04
	**IL-33**	23.00 ± 1.52	14.16 ± 0.39	9.17 ± 0.28
	**IFNG**	11.08 ± 0.25	8.21 ± 0.25	3.20 ± 0.04
	**PDGFB**	1.86 ± 0.05	1.81 ± 0.03	1.33 ± 0.02
	**PDGFC**	81.33 ± 4.46	30.12 ± 1.72	14.60 ± 0.64
	**PDGFD**	17.46 ± 1.04	8.75 ± 0.81	1.97 ± 0.33

	**TNF**	35.20 ± 1.03	20.44 ± 0.28	4.89 ± 0.20
	**WNT3a**	74.97 ± 3.50	7.84 ± 0.27	19.04 ± 1.92
**Cytokine Receptors**	**C3aR1**	12.75 ± 1.70	1.32 ± 0.09	4.20 ± 0.24
	**C5aR1**	971.77 ± 37.41	266.91 ± 4.40	464.74 ± 8.99
	**CCR1**	78.81 ± 1.56	18.73 ± 0.36	66.29 ± 1.88
	**CCR2**	29.38 ± 0.19	15.07 ± 0.38	10.61 ± 0.26
	**CCR3**	90.32 ± 1.78	7.43 ± 0.13	81.21 ± 1.62
	**CCR4**	7.12 ± 0.25	6.84 ± 0.27	4.63 ± 0.26
	**CCR5**	0.72 ± 0.48	1.22 ± 0.05	1.23 ± 0.05
	**CCR6**	52.95 ± 1.99	30.34 ± 1.01	87.05 ± 1.92
	**CCR7**	6.02 ± 0.08	16.68 ± 0.32	52.72 ± 1.16
	**CCR8**	25.09 ± 2.21	15.60 ± 2.60	4.01 ± 0.35
	**CCR10**	3.76 ± 0.07	4.54 ± 0.07	4.13 ± 0.09
	**CSF1R**	289.51 ± 9.43	19.75 ± 0.61	183.69 ± 7.07
	**CSF2R**	17.84 ± 0.51	19.80 ± 0.75	16.07 ± 0.14
	**CX3CR1**	3.48 ± 0.14	1.92 ± 0.05	2.32 ± 0.87
	**CXCR1**	125.37 ± 1.43	30.01 ± 0.76	42.76 ± 1.89
	**CXCR2**	7.39 ± 0.40	2.99 ± 0.36	1.38 ± 0.05
	**CXCR3**	13.50 ± 0.53	4.67 ± 0.16	8.93 ± 0.36
	**CXCR4**	418.13 ± 16.21	4.74 ± 0.62	14.42 ± 0.33
	**CXCR5**	80.52 ± 3.31	29.73 ± 0.92	16.87 ± 1.13
	**CXCR6**	3.52 ± 0.07	3.57 ± 0.10	3.24 ± 0.09
	**FZD1**	74.37 ± 0.63	20.22 ± 0.68	22.89 ± 0.19
	**FZD4**	77.61 ± 3.51	17.04 ± 0.44	40.89 ± 0.79
	**IL1R**	54.34 ± 1.36	68.32 ± 2.25	25.43 ± 1.17
	**IL3R**	4.16 ± 0.08	1.30 ± 0.04	1.58 ± 0.02
	**IL4R**	2.49 ± 0.14	2.19 ± 0.06	1.22 ± 0.06
	**IL5R**	4.48 ± 0.15	3.55 ± 0.23	1.85 ± 0.23
	**IL6R**	66.15 ± 2.37	2.66 ± 0.06	6.28 ± 0.11
	**IL7R**	3.94 ± 0.08	1.18 ± 0.01	1.64 ± 0.07
	**IL8R**	0.98 ± 0.01	1.03 ± 0.02	1.04 ± 0.01
	**IL10R**	95.25 ± 1.84	30.97 ± 1.80	31.33 ± 1.82
	**IL15R**	17.96 ± 0.43	2.05 ± 0.34	5.75 ± 0.15
	**IL17RA**	18.26 ± 0.49	1.91 ± 0.05	11.42 ± 0.20
	**IL17RB**	58.53 ± 2.25	10.96 ± 0.33	41.77 ± 1.59
	**IL17RD**	73.38 ± 2.22	8.01 ± 0.39	26.37 ± 0.98

	**IL18R**	20.27 ± 0.73	1.16 ± 0.04	3.39 ± 0.06
	**IL19R**	2.42 ± 0.16	1.74 ± 0.16	2.81 ± 0.30
	**IL23R**	2.01 ± 0.12	1.58 ± 0.20	0.66 ± 0.06
	**IL33R**	3.36 ± 0.12	3.81 ± 0.75	5.47 ± 0.53
	**IFNGR**	519.36 ± 14.28	81.58 ± 1.35	52.89 ± 2.34
	**PDGFRA**	203.32 ± 7.27	101.20 ± 3.85	16.40 ± 0.92
	**PDGFRB**	2.75 ± 0.05	2.56 ± 0.02	2.52 ± 0.03
	**TIE2**	1.92 ± 0.08	1.15 ± 0.07	1.12 ± 0.01
	**TNFR**	16.63 ± 0.99	6.64 ± 0.12	3.95 ± 0.14
**Miscellaneous**	**BCL2**	13.49 ± 0.46	10.32 ± 0.31	4.10 ± 0.19
	**BCL6**	5.33 ± 0.20	3.39 ± 0.10	3.91 ± 0.14
	**BCLXL**	4.55 ± 0.05	2.96 ± 0.07	3.70 ± 0.10
	**CD14**	23.76 ± 0.49	10.63 ± 0.32	9.76 ± 0.22
	**CD69**	2.22 ± 0.06	1.80 ± 0.03	1.59 ± 0.05
	**CD83**	4.33 ± 0.12	2.93 ± 0.08	2.02 ± 0.02
	**COX2**	12.74 ± 0.53	9.43 ± 0.35	5.90 ± 0.26
	**INOS**	103.06 ± 2.97	92.65 ± 1.94	58.85 ± 3.18
	**LTB**	36.10 ± 0.87	19.16 ± 0.26	17.64 ± 0.63
	**MTOR**	19.12 ± 0.50	8.19 ± 0.35	4.85 ± 0.14
	**SAA**	82.73 ± 1.60	30.28 ± 0.75	21.26 ± 0.42
	**SYK**	26.55 ± 0.84	11.86 ± 0.47	9.83 ± 0.26
	**TLR1**	16.08 ± 0.31	5.17 ± 0.07	5.06 ± 0.21
	**TLR2**	1.86 ± 0.04	5.56 ± 0.17	3.87 ± 0.15
	**TLR3**	11.53 ± 0.44	18.56 ± 0.64	8.38 ± 0.55
	**TLR4**	77.24 ± 3.09	37.44 ± 0.12	30.49 ± 0.42
	**TLR5**	13.81 ± 0.39	10.93 ± 0.29	8.90 ± 0.18
	**TLR6**	7.57 ± 0.22	4.49 ± 0.07	2.27 ± 0.09
	**TLR7**	7.30 ± 0.21	4.30 ± 0.19	2.28 ± 0.08
	**TLR8**	7.75 ± 0.22	4.60 ± 0.08	4.09 ± 0.13
	**TLR10**	15.14 ± 0.43	8.98 ± 0.15	7.00 ± 0.17
	**TRIF**	17.81 ± 0.66	11.85 ± 0.20	9.09 ± 0.35

[A] The gene expression profiling files are available from the GEO database (accession numbers GSE 75518 and GSE 78162).

[B] All primers were designed based on human CDS of targets found in NCBI database and validated using human universal cDNA. Primers were confirmed to exclusively detect species-specific cDNA.

[C] $CD14^+$ monocytes were treated with LPS (50 ng/ml), HMGB1 (50 ng/ml) or nab-PXL (30 nM).

[D] The mean of fold-change calculated based on β-actin normalized Ct values obtained in triplicate from each treatment group.
