# Supplementary material for: Lymphatic endothelial progenitors originate from plastic myeloid cells activated by toll-like receptor-4
Source: PLoS One. 2017 Jun 9;12(6):e0179257. doi: 10.1371/journal.pone.0179257 (PMC5466303; doi:10.1371/journal.pone.0179257)
Supplement: S4 Table — (PDF) [file pone.0179257.s007.pdf]

S4 Table. TLR4-activated lymphatic reprogramming of bone marrow myeloid cells derived from C57BL/6 mice.

Gene	Mouse 1[A]	Mouse 2	Mouse 3	Mouse 4	Average
**Ccl2**	18.72 ± 0.78	22.30 ± 1.70	23.42 ± 1.86	12.56 ± 0.22	19.25 ± 4.89
**Ccl19**	3.73 ± 0.01	2.36 ± 0.07	3.59 ± 0.02	4.10 ± 0.01	3.45 ± 0.75
**Ccl20**	52.00 ± 1.08	42.67 ± 0.44	54.57 ± 0.76	41.93 ± 0.44	47.79 ± 6.44
**Ccr1**	5.82 ± 0.20	9.44 ± 1.08	5.91 ± 0.39	8.72 ± 0.03	7.47 ± 1.88
**Ccr3**	39.53 ± 0.27	37.02 ± 0.38	40.56 ± 2.11	35.02 ± 0.24	38.03 ± 2.50
**Ccr6**	46.54 ± 1.13	61.41 ± 1.28	36.97 ± 2.56	41.79 ± 0.58	46.68 ± 10.57
**Csf1r**	31.58 ± 1.20	26.27 ± 0.36	34.33 ± 1.43	33.14 ± 0.80	31.33 ± 3.56
**Cxcl1**	48.18 ± 0.83	66.51 ± 1.61	43.57 ± 0.75	53.10 ± 1.66	52.84 ± 9.91
**Cx3cl1**	9.17 ± 0.38	11.78 ± 0.73	7.73 ± 0.08	8.42 ± 1.16	9.28 ± 1.77
**Cx3cr1**	28.05 ± 0.10	42.22 ± 0.15	33.16 ± 1.49	32.72 ± 1.70	34.04 ± 5.92
**CD14**	36.64 ± 0.63	34.64 ± 3.94	34.06 ± 0.12	37.40 ± 0.26	35.69 ± 1.59
**CD33**	18.70 ± 0.26	18.51 ± 0.51	22.79 ± 0.47	21.71 ± 0.30	20.43 ± 2.15
**CD105**	24.25 ± 0.08	25.38 ± 0.62	35.38 ± 0.12	24.94 ± 0.69	27.49 ± 5.28
**CD133**	6.55 ± 2.08	14.83 ± 0.31	6.15 ± 0.04	5.12 ± 0.16	8.16 ± 4.49
**CD146**	1.48 ± 0.03	10.98 ± 0.53	2.61 ± 0.02	1.70 ± 0.01	4.19 ± 4.55
**Cdx-2**	2.72 ± 0.01	2.69 ± 0.08	3.08 ± 0.04	2.41 ± 0.11	2.73 ± 0.27
**C5ar1**	85.34 ± 1.48	79.72 ± 4.14	84.19 ± 2.33	77.50 ± 2.95	81.69 ± 3.70
**E2f1**	1.46 ± 0.02	5.51 ± 0.23	2.10 ± 0.10	1.53 ± 0.08	2.65 ± 1.93
**Hoxa4**	30.70 ± 0.21	47.67 ± 0.07	39.70 ± 1.65	28.78 ± 1.59	36.71 ± 8.72
**Il-10**	69.60 ± 2.65	87.68 ± 6.67	66.62 ± 4.15	67.88 ± 0.24	72.95 ± 9.90
**Il-15**	83.35 ± 3.18	90.83 ± 1.26	90.52 ± 1.25	79.77 ± 4.97	86.12 ± 5.46
**Ifngr1**	16.91 ± 0.23	18.79 ± 1.88	15.14 ± 0.05	18.64 ± 0.32	17.37 ± 1.71
**Irf7**	9.85 ± 0.03	16.35 ± 0.68	7.60 ± 0.29	12.55 ± 0.04	11.59 ± 3.77
**Itga9**	5.38 ± 0.28	10.41 ± 0.07	4.83 ± 0.20	2.80 ± 0.02	5.86 ± 3.23
**Lyve-1**	16.51 ± 0.06	24.43 ± 0.76	19.12 ± 1.06	14.12 ± 1.99	18.55 ± 4.42
**Maf**	1.79 ± 0.07	3.98 ± 0.25	1.97 ± 0.06	1.72 ± 0.03	2.37 ± 1.08
**Mafb**	11.88 ± 0.45	9.16 ± 0.13	10.10 ± 1.22	11.24 ± 0.08	10.60 ± 1.21
**Notch1**	1.26 ± 0.02	5.24 ± 0.09	2.20 ± 0.13	2.38 ± 0.03	2.77 ± 1.72
**Pax6**	17.43 ± 1.21	18.78 ± 0.78	16.56 ± 0.11	14.68 ± 0.41	16.86 ± 1.72
**Pecam-1**	3.27 ± 0.01	3.30 ± 0.40	2.72 ± 0.05	2.83 ± 0.01	3.03 ± 0.30
**Pdpn**	117.82 ± 2.86	123.70 ± 3.86	127.69 ± 5.75	122.79 ± 0.43	123.00 ± 4.06
**Saa**	14.88 ± 0.31	16.17 ± 0.06	16.28 ± 0.17	14.27 ± 0.05	15.40 ± 0.99
**Six1**	54.95 ± 0.76	42.22 ± 3.50	55.12 ± 4.38	50.22 ± 0.70	50.63 ± 6.05
**Syk**	22.79 ± 0.16	34.16 ± 3.89	20.04 ± 0.28	28.64 ± 0.40	26.41 ± 6.29
**Tead2**	100.21 ± 5.21	77.28 ± 4.01	86.53 ± 0.90	112.61 ± 1.56	94.16 ± 15.49
**Tie2**	3.00 ± 0.09	5.05 ± 0.12	2.93 ± 0.13	2.47 ± 0.24	3.36 ± 1.15

**Tlr2**	11.08 ± 0.23	16.01 ± 1.49	17.16 ± 2.89	18.07 ± 0.50	15.58 ± 3.12
**Tlr4**	11.35 ± 0.20	11.79 ± 1.90	16.63 ± 0.35	14.17 ± 0.10	13.49 ± 2.44
**Vegfr-1**	4.84 ± 0.12	19.79 ± 1.03	10.10 ± 0.31	5.25 ± 0.31	10.00 ± 6.95
**Vegfr-2**	26.27 ± 0.46	40.96 ± 1.56	31.57 ± 0.88	27.97 ± 0.97	31.69 ± 6.56
**Vegfr-3**	9.09 ± 0.06	24.26 ± 0.50	11.76 ± 0.29	12.25 ± 0.21	14.34 ± 6.76
**Vegfc**	90.20 ± 0.63	104.90 ± 6.54	92.66 ± 6.73	85.06 ± 7.35	93.21 ± 8.42
**Vegfd**	2.73 ± 0.09	4.22 ± 0.22	2.95 ± 0.06	3.39 ± 0.28	3.32 ± 0.66

[A] Results are presented as fold-increases in nab-PXL treated monocytes relative to monocytes treated only with CSF1. Each target was analyzed in triplicate and mean ± SEM is presented.
