# Supplementary material for: Lymphatic endothelial progenitors originate from plastic myeloid cells activated by toll-like receptor-4
Source: PLoS One. 2017 Jun 9;12(6):e0179257. doi: 10.1371/journal.pone.0179257 (PMC5466303; doi:10.1371/journal.pone.0179257)
Supplement: S5 Table — (PDF) [file pone.0179257.s008.pdf]

S5 Table. TLR4-activated lymphatic reprogramming of bone marrow myeloid cells derived from CB-17/SCID mice.

Gene	Mouse 1[A]	Mouse 2	Mouse 3	Average
**Ccl2**	37.32 ± 2.56	29.14 ± 0.14	23.68 ± 0.70	30.05 ± 6.87
**Ccl19**	1.72 ± 0.07	1.99 ± 0.05	2.30 ± 0.91	2.00 ± 0.29
**Ccl20**	63.88 ± 5.00	87.50 ± 5.14	80.48 ± 3.15	77.29 ± 12.13
**Ccr1**	9.75 ± 0.48	10.00 ± 0.59	21.34 ± 2.61	13.70 ± 6.62
**Ccr3**	35.19 ± 2.76	39.61 ± 3.49	47.02 ± 1.15	40.61 ± 5.98
**Ccr6**	38.75 ± 2.28	29.55 ± 0.29	39.13 ± 1.15	35.81 ± 5.42
**Csf1r**	8.95 ± 0.70	8.62 ± 1.64	16.57 ± 0.32	11.38 ± 4.50
**Cxcl1**	35.76 ± 0.70	38.13 ± 5.59	47.68 ± 1.64	40.52 ± 6.31
**Cx3cl1**	8.88 ± 0.26	8.73 ± 1.07	10.97 ± 0.11	9.53 ± 1.25
**Cx3cr1**	27.35 ± 2.68	39.04 ± 7.04	17.09 ± 0.34	27.83 ± 10.98
**CD14**	30.60 ± 0.90	36.90 ± 1.63	27.25 ± 2.53	31.58 ± 4.90
**CD33**	8.64 ± 0.55	7.20 ± 0.60	6.79 ± 0.56	7.54 ± 0.97
**CD105**	40.81 ± 1.80	30.26 ± 3.26	43.05 ± 6.31	38.04 ± 6.83
**CD133**	8.21 ± 0.56	8.29 ± 1.05	9.65 ± 0.28	8.72 ± 0.81
**CD146**	1.99 ± 0.06	2.05 ± 0.46	4.78 ± 0.28	2.94 ± 1.59
**Cdx-2**	0.97 ± 0.27	1.40 ± 0.02	1.36 ± 0.01	1.24 ± 0.24
**C5ar1**	49.36 ± 6.03	35.93 ± 3.52	43.90 ± 2.37	43.07 ± 6.74
**E2f1**	2.69 ± 0.05	1.97 ± 0.20	2.48 ± 0.17	2.38 ± 0.37
**Hoxa4**	13.10 ± 3.55	15.01 ± 4.14	21.91 ± 2.14	16.67 ± 4.63
**Il-10**	37.69 ± 2.22	43.53 ± 6.80	55.35 ± 2.17	45.52 ± 9.00
**Il-15**	75.99 ± 6.70	71.32 ± 9.41	86.60 ± 5.09	77.97 ± 7.83
**Ifngr1**	4.69 ± 0.05	5.26 ± 0.69	10.53 ± 1.44	6.83 ± 3.22
**Irf7**	5.78 ± 0.71	4.44 ± 0.59	7.49 ± 0.07	5.90 ± 1.53
**Itga9**	2.23 ± 0.41	2.84 ± 0.15	3.28 ± 0.30	2.78 ± 0.53
**Lyve-1**	10.25 ± 1.90	9.68 ± 2.26	10.56 ± 0.52	10.16 ± 0.45
**Maf**	4.08 ± 0.08	4.81 ± 0.16	4.25 ± 0.37	4.38 ± 0.38
**Mafb**	16.68 ± 0.41	19.60 ± 3.63	35.26 ± 0.35	23.85 ± 9.99
**Notch1**	2.60 ± 0.33	2.85 ± 0.17	2.22 ± 0.19	2.56 ± 0.32
**Pax6**	11.04 ± 1.19	8.27 ± 0.65	8.20 ± 0.28	9.17 ± 1.62
**Pecam-1**	1.99 ± 0.08	1.84 ± 0.38	3.48 ± 0.12	2.44 ± 0.91
**Pdpn**	102.42 ± 38.70	92.68 ± 9.97	99.04 ± 0.49	98.05 ± 4.95
**Saa**	13.41 ± 0.01	17.10 ± 0.92	22.55 ± 0.22	17.69 ± 4.60
**Six1**	49.92 ± 3.18	59.93 ± 1.47	75.85 ± 1.49	61.90 ± 13.08
**Syk**	14.83 ± 0.73	23.92 ± 0.23	23.11 ± 1.02	20.62 ± 5.03
**Tead2**	77.24 ± 13.56	70.85 ± 9.69	58.15 ± 7.96	68.75 ± 9.72
**Tie2**	2.31 ± 0.06	1.73 ± 0.38	1.67 ± 0.22	1.90 ± 0.35

**Tlr2**	5.24 ± 0.15	4.86 ± 0.05	8.57 ± 0.92	6.22 ± 2.04
**Tlr4**	18.07 ± 0.71	18.51 ± 0.45	29.90 ± 2.20	22.16 ± 6.71
**Vegfr-1**	5.37 ± 0.60	3.17 ± 0.69	5.12 ± 0.28	4.55 ± 1.20
**Vegfr-2**	10.37 ± 0.10	18.84 ± 4.48	27.57 ± 0.27	18.93 ± 8.60
**Vegfr-3**	7.76 ± 0.19	8.04 ± 0.55	12.69 ± 0.31	9.50 ± 2.77
**Vegfc**	98.48 ± 6.75	105.65 ± 9.82	105.08 ± 3.09	103.07 ± 3.99
**Vegfd**	1.91 ± 0.08	3.45 ± 0.42	2.94 ± 0.60	2.77 ± 0.78

[A] Results are presented as fold-increases in nab-PXL treated monocytes relative to monocytes treated only with CSF1. Each target was analyzed in triplicate and mean ± SEM is presented.
